# Reduction of pTau and APP levels in mammalian brain after low-dose radiation

**DOI:** 10.1038/s41598-021-81602-z

**Published:** 2021-01-26

**Authors:** Diego Iacono, Erin K. Murphy, Soundarya S. Avantsa, Daniel P. Perl, Regina M. Day

**Affiliations:** 1 DoD/USU Brain Tissue Repository and Neuropathology Core, Uniformed Services University (USU), Bethesda, MD USA; 2 Department of Neurology, F. Edward Hébert School of Medicine, Uniformed Services University (USU), Bethesda, MD USA; 3 Department of Pathology, F. Edward Hébert School of Medicine, Uniformed Services University (USU), Bethesda, MD USA; 4 The Henry M. Jackson Foundation for the Advancement of Military Medicine (HJF), 4301 Jones Bridge Road, A1036, Bethesda, MD 20814-4799 USA; 5 Complex Neurodegenerative Disorders, National Institute of Neurological Disorders and Stroke, NINDS, NIH, Bethesda, MD USA; 6 Department of Pharmacology and Molecular Therapeutics, Uniformed Services University (USU), Bethesda, MD USA

**Keywords:** Neurology, Neurodegenerative diseases, Therapeutics

## Abstract

Brain radiation can occur from treatment of brain tumors or accidental exposures. Brain radiation has been rarely considered, though, as a possible tool to alter protein levels involved in neurodegenerative disorders. We analyzed possible molecular and neuropathology changes of phosphorylated-Tau (pTau), all-Tau forms, *β*-tubulin, amyloid precursor protein (APP), glial fibrillary acidic protein (GFAP), ionized calcium binding adaptor molecule 1 (IBA-1), myelin basic protein (MBP), and GAP43 in Frontal Cortex (FC), Hippocampus (H) and Cerebellum (CRB) of swine brains following total-body low-dose radiation (1.79 Gy). Our data show that radiated-animals had lower levels of pTau in FC and H, APP in H and CRB, GAP43 in CRB, and higher level of GFAP in H versus sham-animals. These molecular changes were not accompanied by obvious neurohistological changes, except for astrogliosis in the H. These findings are novel, and might open new perspectives on brain radiation as a potential tool to interfere with the accumulation of specific proteins linked to the pathogenesis of various neurodegenerative disorders.

## Introduction

Generally, the central nervous system (CNS), and more specifically the brain, can be the target organ for radiation for medical purposes (e.g. treatments for primary brain tumors or palliative cures for cerebral metastatic neoplasia) or be one of the vital organs injured by nuclear power accidents, accidental nuclear contamination or nuclear war/attack^1–3^. Although brain radiation has been demonstrated to be useful in treating different types of brain tumors, especially when combined with other types of therapies^4,5^, short- and long-term molecular and neuropathological effects of radiation on normal brain tissue are not completely understood^6–9^.

Radiation-induced brain injury due to fractionated or single high-dose radiotherapy can generate a complex series of acute^10–12^ and chronic changes^13,14^, where the resulting clinical severity and therapeutic outcomes depend on the nature of the brain pathology involved (primary brain tumor vs. brain metastases), general medical conditions (older vs. younger patients), radiation techniques used (fractionated vs. single dose), requirement of isolated or combined therapies (isolated radiotherapy vs. antiblastic + radiation therapy), and total dose of *γ*-rays administered (low- vs. high-dose). Clinical evidence shows that brain radiation can generate significant and often delayed (> 6 months/years) neurological consequences (e.g. cognitive impairment, mood disorders, sleep disorders) that go beyond the direct effects of radiation on brain tumors^15^. Even targeted radiation treatments for brain tumors may have detrimental consequences on the normal tissue contiguous to the pathological targeted lesions^7,16,17^. Frequently, patients that suffer the most from these neurological consequences are children who underwent brain radiation as part of their treatment for either primary or secondary brain tumors^18–23^. The majority of the previous neuropathological studies have principally focused on brain tissues obtained from rodents^24–26^ or from surgically resected human specimens after radiation and chemotherapy treatments^27^. As an alternative, simplified experimental models have employed neuronal or glial cell cultures^28–30^. Because higher cognitive and behavioral capacities have been demonstrated to be among the long-term effects of brain radiation in humans^31^, the use of higher order mammals are needed to adequately model radiation effects on the human brain.

Systematic neuropathological investigations using modern molecular protocols such as protein expression level quantification through Western blotting [WB] combined with more sensitive immunohistochemistry-based neurohistological techniques (polymers-based immunohistochemistry method) to examine the possible consequences of low-dose total body/brain radiation exposure (e.g. ~ 2.0 Gy) on large mammalian brains under normal anatomo-physiological conditions are currently lacking^32–34^. Swine have much more similar metabolic, cognitive and behavioral capacities to humans than rodents. Additionally, swine have been shown to be a valuable model to study different aspects of radiation either in terms of radiation effects or radiation countermeasures due to their anatomical and physiological similarity to humans^35–37^. The similarity of swine physiology to humans as well as their current use for the study of radiation effects led us to hypothesize that this model would be informative for the study of radiation effects on the brain.

The primary goal of our study was to determine whether a single exposure to low-dose radiation on a large mammalian brain under normal anatomo-physiological conditions could produce changes at the molecular level that could potentially generate a scientific rationale for the future applications of low-dose brain radiation as a preventive or therapeutic tool for different neurodegenerative disorders in humans. In particular, very little is known about the possible effects of low-dose brain radiation in the context of misfolded protein mechanisms involved in the various pathogenetic processes of human neurodegenerative disorders such as tauopathies, synucleinopathies or prion diseases.

In this study, we chose to perform a set of molecular analyses examining protein expression levels across different neuroanatomical regions and possible corresponding neuropathological effects in large normal mammalian brains 4-weeks following a single, 1.79 Gy dose, of total-body radiation. We hypothesized that low-dose brain radiation could interfere with proteins normally expressed in the brain which might prove to be beneficial in those CNS conditions characterized by pathological accumulations of extra- or intra-cellular misfolded proteins such as *β*-amyloid and hyperphosphorylated-Tau lesions in Alzheimer’s disease (AD), α-synuclein-positive Lewy bodies in Parkinson’s disease (PD), or prion misfolded protein in Creutzfeldt–Jakob disease (CJD)^38^. Our hypothesis was previously proposed by only a very small number of investigations that mainly used transgenic rodents or cell cultures^25,39,40^.

Specifically, we aimed:

(1) to identify early (4 weeks post-radiation) molecular brain alterations such as changes of soluble proteins expression levels [e.g. phosphorylated-Tau (pTau), all-forms of Tau, amyloid precursor protein (APP), glial fibrillary acidic protein (GFAP), ionized calcium binding adaptor molecule 1 (IBA-1), myelin basic protein (MBP) and neosynaptogenic markers (e.g. GAP43)], in large normal mammalian brains (swine brains) after a low-dose total-body radiation;

(2) to determine whether different regions of a large mammalian brain (frontal cortex [FC], hippocampus [H] and cerebellum [CRB]) reacted to radiation by differential expression levels of those proteins when measured at an identical post-radiation time-point (4 weeks);

(3) to determine whether captopril—a commonly prescribed angiotensin converting enzyme (ACE) inhibitor for the treatment of hypertension—which was shown to provide improvement in some of the post-radiation neuropathological and hematological side effects in mice (brain microhemorrhages, hematopoietic injuries)^41^—might also have some modifying effects when administered to larger mammalian brains.

Our study represents a specific brain-targeted investigation that was part of a larger DoD-funded project aiming to analyze the possible beneficial effects of captopril in larger animals on a series of hematological consequences of total body radiation. Previous studies, in fact, have shown that captopril when administered after radiation was able to drastically reduce some of the post-radiation hematological effects of a single high-dose (8.25 Gy) of total body radiation in rodents^42^.

## Results

### Post-radiation general conditions

Based on the approved criteria for early euthanasia (recumbence with failure to gain standing posture, severe lethargy, and/or ataxia), two animals from the Radiation + Vehicle group were euthanized prior to the study end point due to radiation injuries, one at 19 days (for lethargy) and another at 20 days (for lethargy) post-radiation. The remaining animals, sham- (SH-) and radiated (RAD-) animals were euthanized at 33–35 days post-radiation and demonstrated no overt signs of neurological or systemic (fever) illness. No significant difference in body weight was demonstrated between SH- and RAD-animals across the entire study time course (baseline: SH = 27.5 vs RAD = 22.9; Day 16: SH = 32.6 vs RAD = 29.4; Day 30: SH = 35.6 vs RAD = 35.9; weights expressed in pounds) (see Supplementary Fig. 1).

**Figure 1:**
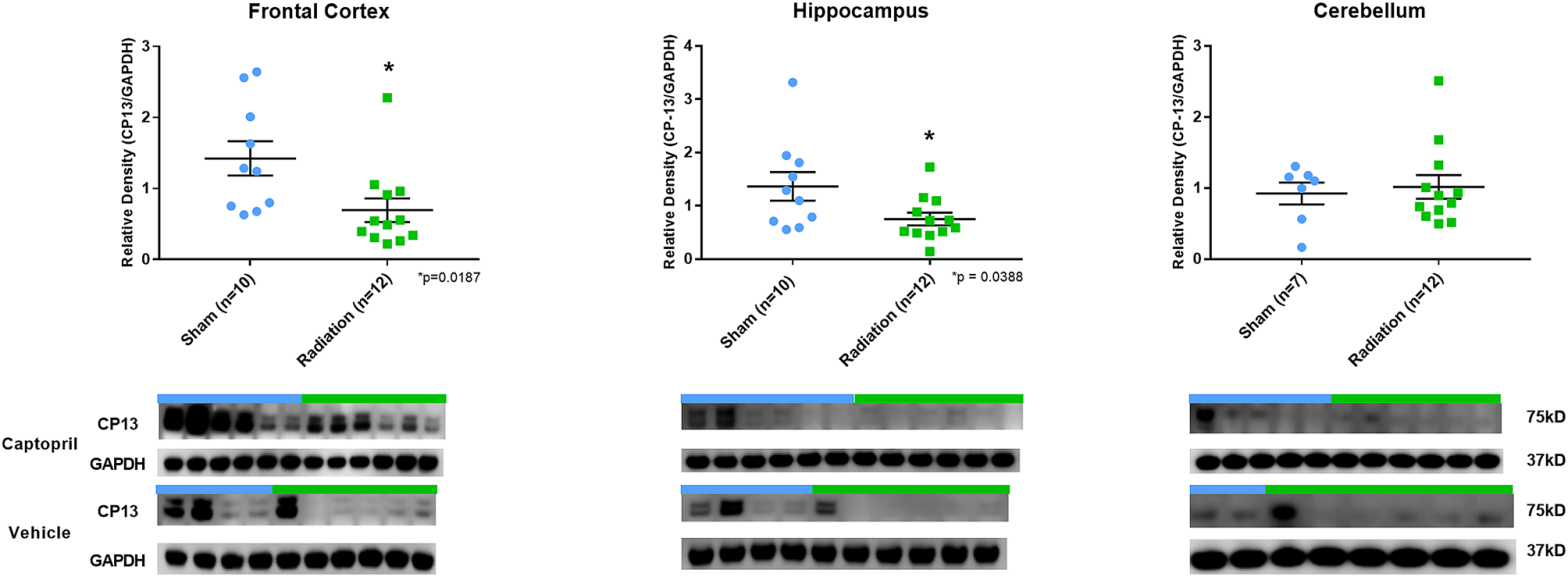
Phosphorylated-Tau expression in brain following total body radiation. Histograms representing the densitometric ratio of levels of pTau (CP13) with respect to GAPDH as measured in the frontal cortex, hippocampus and cerebellum in the brains of Gottingen mini-pigs 30-days after total body radiation (1.79 Gy of Cobalt [^60^Co]) with representative western blots^#^ for sham and radiation exposed animals treated with captopril or vehicle. There was no statistical difference between captopril or vehicle treated groups, so data was pooled for each group represented in histograms above. *indicates *p* values < 0.05 as determined by 2-tailed, unpaired, t-tests. Error bars represent standard error of the mean (SEM). ^#^for full length blots for each antibody, see Supplementary Fig. S11-S30.

### Effect of captopril across sham- and radiated-animal groups

We found no statistically significant differences between captopril and vehicle administration in either SH- or RAD–animal groups across all examined brain regions in terms of protein expression levels (see Supplementary Figs. 2–7). Due to these preliminary outcomes, captopril and vehicle animals were pooled together in two groups: sham-animal group (SH-group; vehicle + captopril sham animals) and radiated-animal group (RAD-group; vehicle + captopril radiated animals). See “Methods” section for more details on the statistical analyses performed.

**Figure 2:**
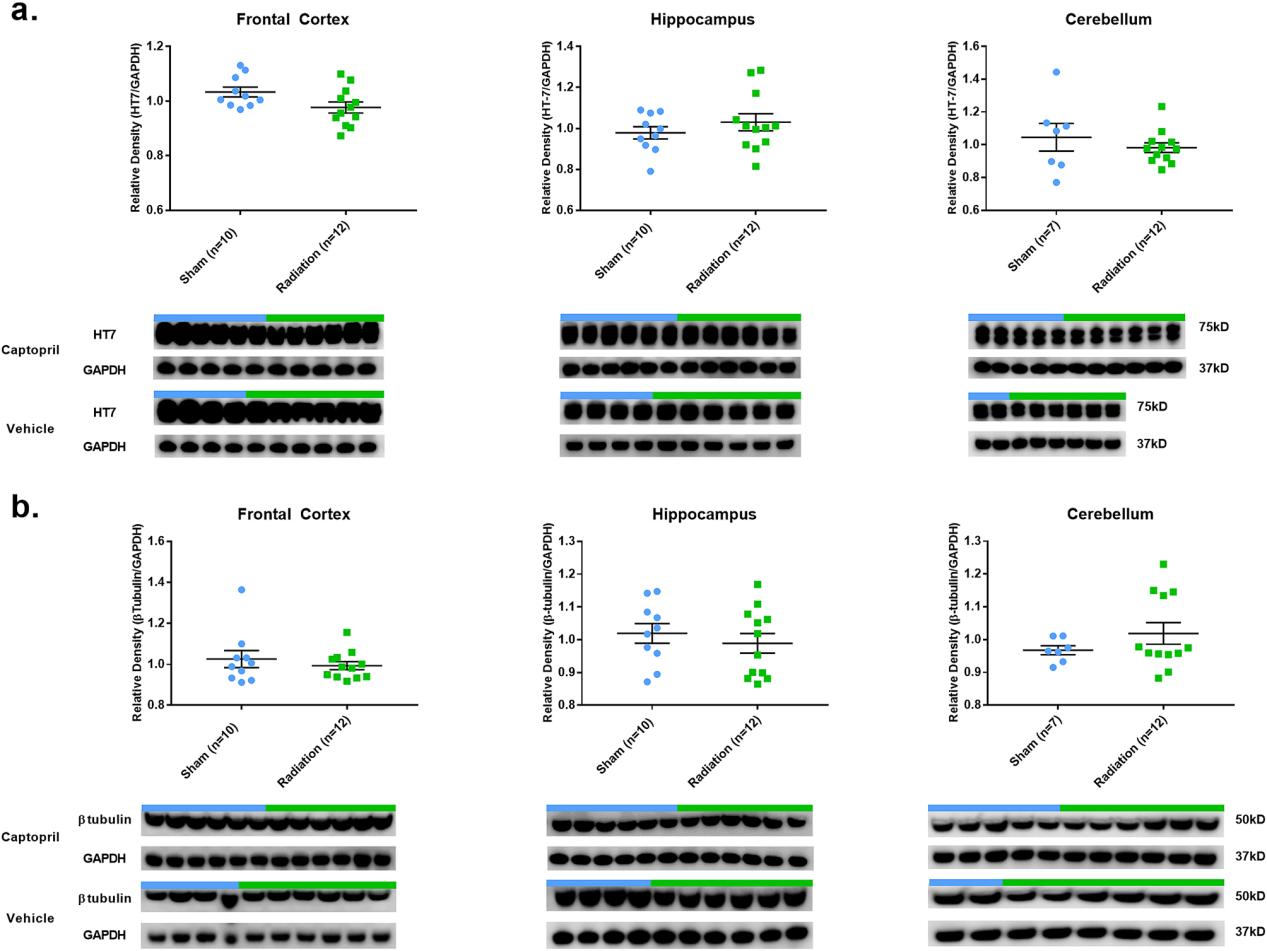
Total-Tau and β-tubulin expression in brain following total body radiation. Histograms representing the densitometric ratio of levels of **(a)** total Tau (HT7) and **(b)** *β*-tubulin with respect to GAPDH as measured in the frontal cortex, hippocampus and cerebellum in the brains of Gottingen mini-pigs 30-days after total body radiation (1.79 Gy of Cobalt [^60^Co]) with representative western blots^#^ for sham and radiation exposed animals treated with captopril or vehicle. There was no statistical difference between captopril or vehicle treated groups, so data was pooled for each group represented in histograms above. *indicates *p* values < 0.05 as determined by 2-tailed, unpaired, t-tests. Error bars represent standard error of the mean (SEM). ^#^for full length blots for each antibody, see Supplementary Fig. S11-S30.

### Protein expression levels

**Figure 3:**
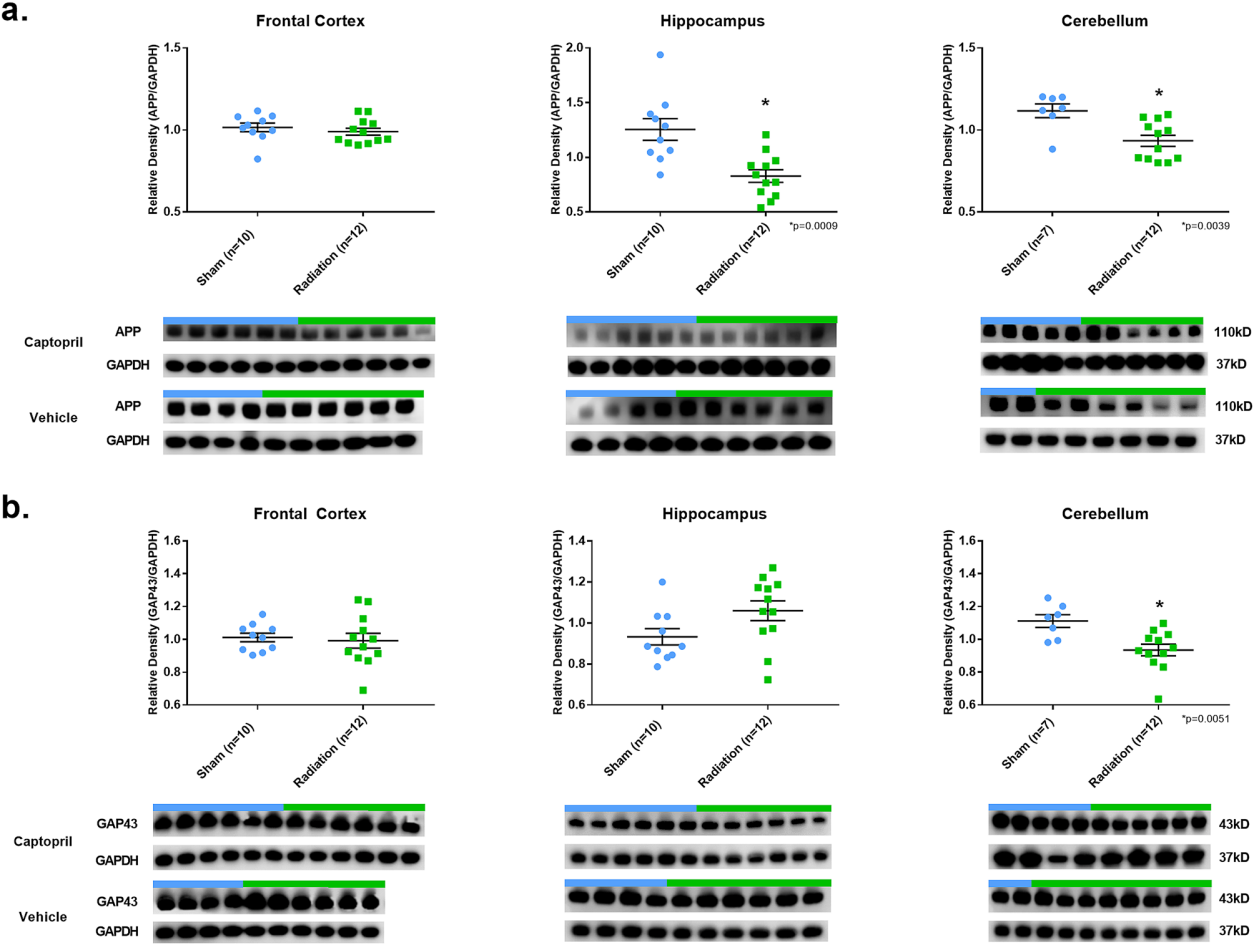
APP and GAP43 expression in brain following total body radiation. Histograms representing the densitometric ratio of levels of APP (**a**) and GAP43 (**b**) with respect to GAPDH as measured in the frontal cortex, hippocampus and cerebellum in the brains of Gottingen mini-pigs 30-days after total body radiation (1.79 Gy of Cobalt [^60^Co]) with representative western blots^#^ for sham and radiation exposed animals treated with captopril or vehicle. There was no statistical difference between captopril or vehicle treated groups, so data was pooled for each group represented in histograms above. * indicates *p* values < 0.05 as determined by 2-tailed, unpaired, t-tests. Error bars represent standard error of the mean (SEM). ^#^for full length blots for each antibody, see Supplementary Fig. S11-S30.

Our results show that phosphorylated-Tau levels (CP13) were lower in RAD-versus SH-animals in the FC (*p* = 0.0187, df = 20) and H (*p* = 0.0388, df = 20) regions (Fig. 1). Moreover, HT7 (all-Tau forms) and *β*-tubulin levels did not differ across any of the examined neuroanatomical regions (FC, H, CRB) (Fig. 2). Furthermore, APP protein expression level was lower in RAD-versus SH-animals in the H (*p* = 0.0009, df = 20) and CRB region (*p* = 0.0039, df = 17) (Fig. 3a). In addition, GAP43 expression level in the CRB was lower in RAD-versus SH-animals as well (*p* = 0.0051, df = 17) (Fig. 3b).

**Figure 4:**
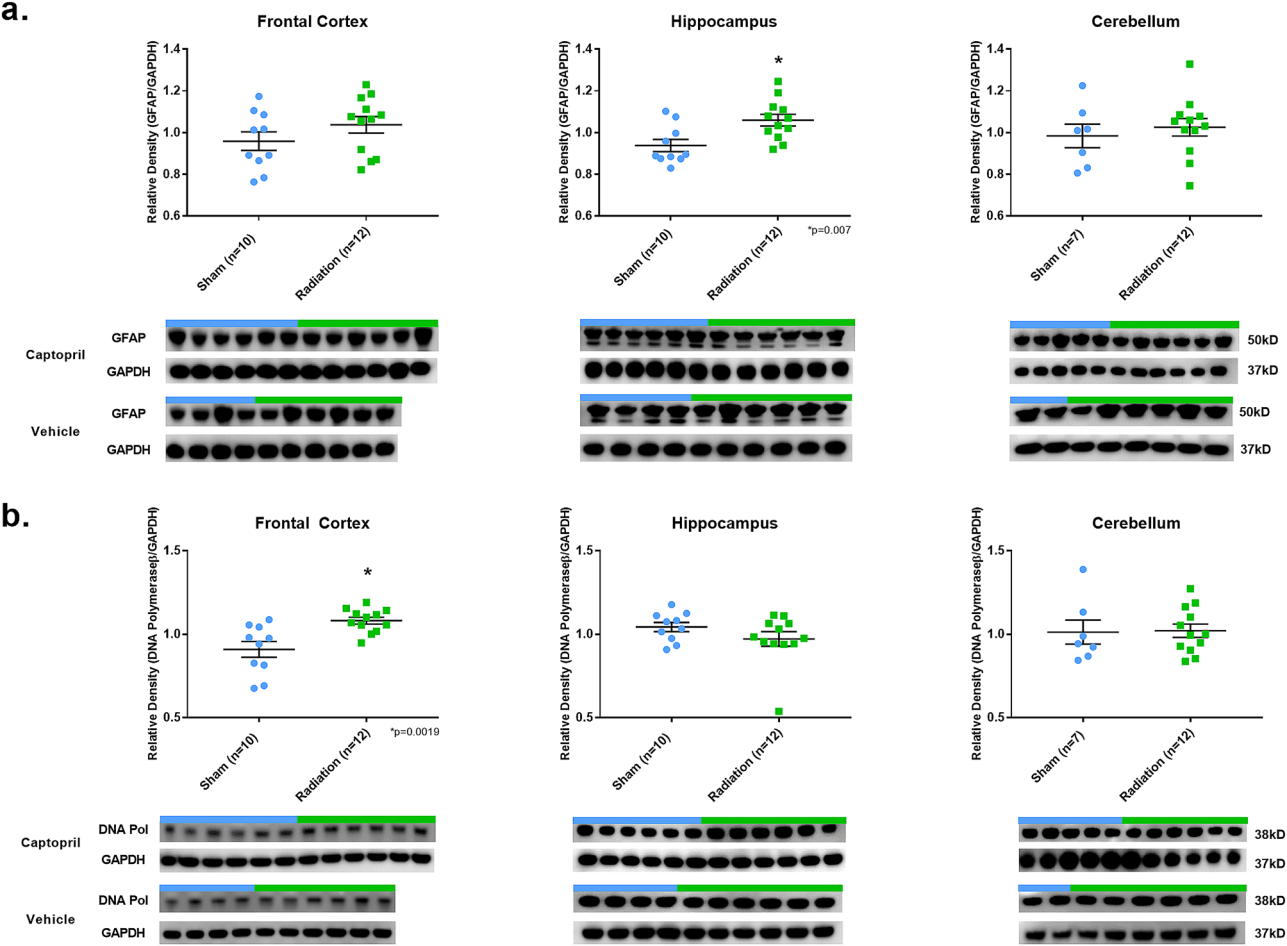
GFAP and DNA Polymerase-β expression in brain following total body radiation. Histograms representing the densitometric ratio of levels of GFAP (**a**) and DNA polymerase-*β* (**b**) with respect to GAPDH as measured in the frontal cortex, hippocampus and cerebellum in the brains of Gottingen mini-pigs 30-days after total body radiation (1.79 Gy of Cobalt [^60^Co]) with representative western blots^#^ for sham and radiation exposed animals treated with captopril or vehicle. There was no statistical difference between captopril or vehicle treated groups, so data was pooled for each group represented in histograms above. *indicates *p* values < 0.05 as determined by 2-tailed, unpaired, *t*-tests. Error bars represent standard error of the mean (SEM). ^#^for full length blots for each antibody, see Supplementary Fig. S11-S30.

By contrast, GFAP expression level was higher in RAD-versus SH-animals in the H region (*p* = 0.007, df = 20). In addition, only in the FC region, an increase of DNA polymerase-*β* in RAD-versus SH-animals (*p* = 0.0019, df = 20) was found (Fig. 4).

IBA-1 and MBP expression levels did not differ between RAD-versus SH-animals in any of the examined brain regions. Finally, the measured expression levels of all phosphorylation-related enzymes (GSK3*β*, pGSK3*β* [Y216], PP2A-*βα*) did not differ in RAD- versus SH-animals in any examined region, except in CRB for GSK3*β* (Supplementary Figs. 8–10).

### Immunohistochemistry and neurohistology outcomes

**Figure 5:**
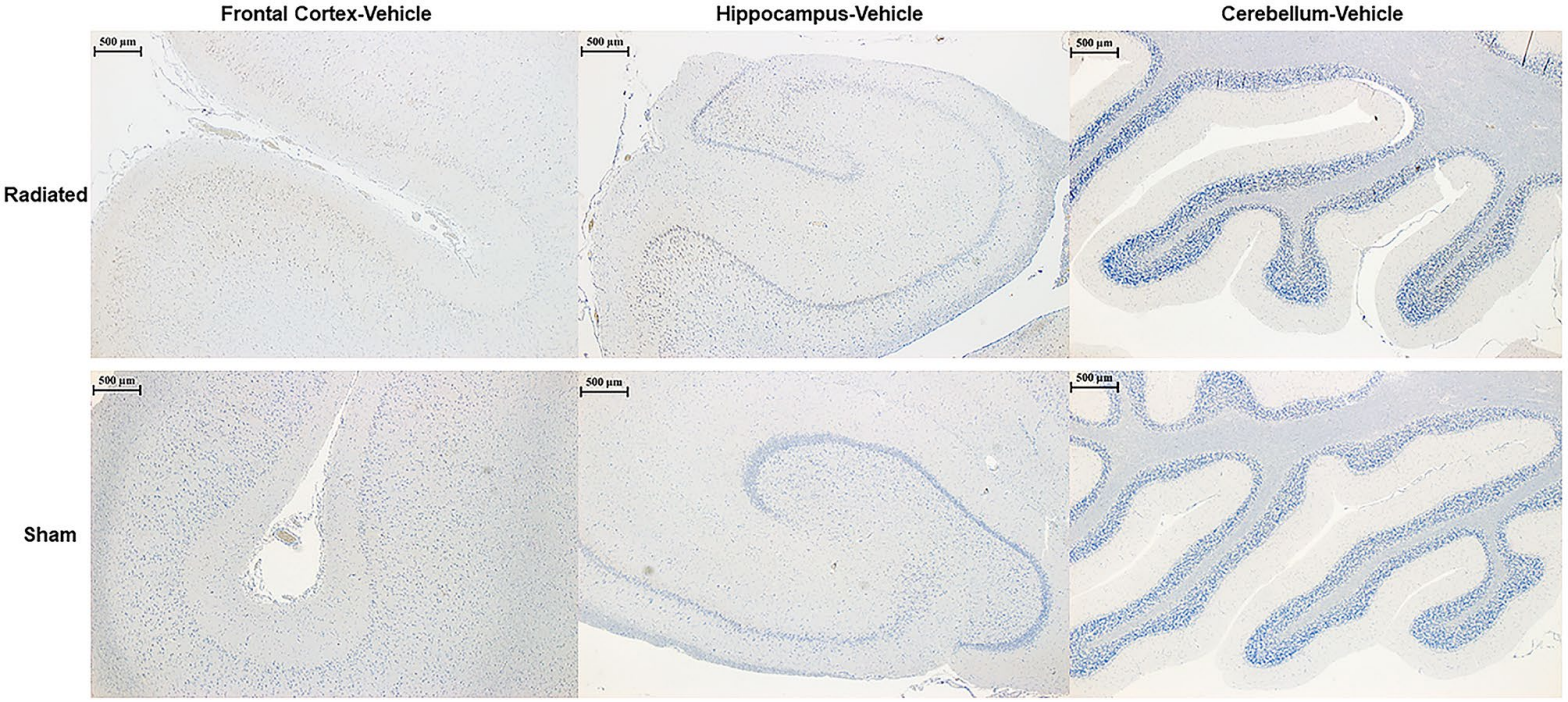
CP13 Immunohistochemistry across Different Regions of Swine Brains after 4 weeks following low-dose post-radiation. The figure shows Frontal Cortex, Hippocampus and Cerebellar Cortex of a Radiated and a Sham vehicle treated animal immunostained for pTau using CP13 antibody. To notice, no differences were present at histological level between Radiated versus Sham animals across any of the anatomical region considered. The digital photographs were taken using a bright-light microscope (Zeiss ImagerA2) with a 20 × objective.

**Figure 6:**
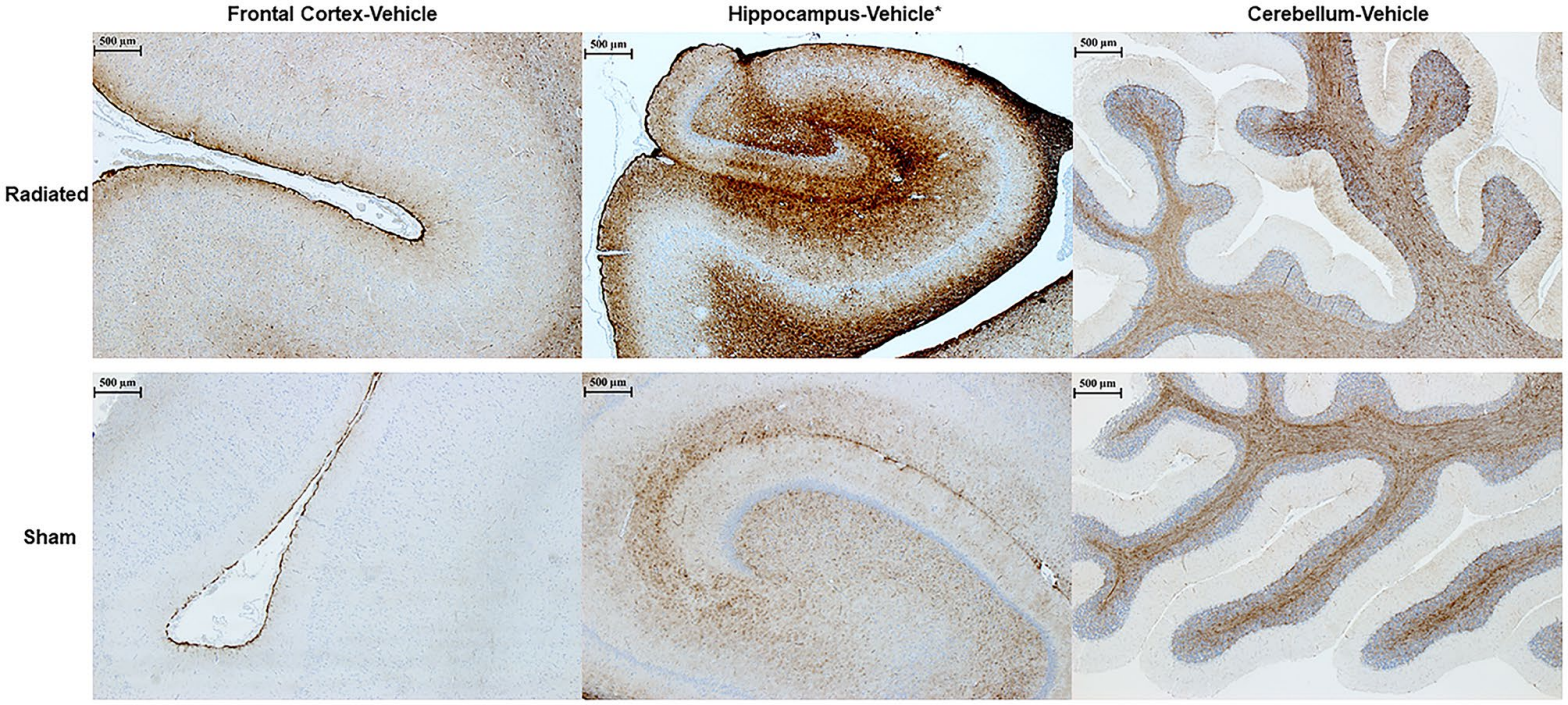
GFAP Immunohistochemistry across Different Regions of Swine Brain after 4 weeks following low-dose post-radiation. The figure shows Frontal Cortex, Hippocampus and Cerebellar Cortex of a Radiated and a Sham vehicle treated animal immunostained for glial fibrillary acidic protein (GFAP), a marker for astroglial cells. *To notice the presence of the astroglial response in the Hippocampus (H), specifically in the peri-dental gyrus area, of the Radiated versus Sham animal. The digital photographs were taken using a bright-light microscope (Zeiss ImagerA2) with a 20 × objective.

Immunohistochemistry protocols for CP13, HT7, APP, IBA-1, GFAP and MBP did not show any pathological accumulation of insoluble intra- (e.g. pTau neurofibrillary tangles-like lesions) or extra-cellular proteins (e.g. APP-positive lesions) in RAD-versus SH-animals (Fig. 5). However, initial astroglial reaction (as assessed by HE stain and GFAP immunostain) was present in the H of RAD-versus SH-animals (Fig. 6), specifically in the peri-dentate gyrus (peri-DG) area. HE, LFB, and CV stains did not show any intraparenchymal or necrotic-ischemic vascular lesions (including microhemorrhages) (HE), obvious myelin loss (LFB stain) or any evident chromosomal aberration, nuclear or perinuclear damage (CV stain) in neurons or glial cells.

## Discussion

While most research investigations on radiation have focused on minimizing the long-term neurological consequences of specific radiotherapy protocols used in neuro-oncology (using low-dose fractionated and targeted radiation for primary brain tumors) or on looking for prophylactic tools to prevent or minimize systemic injurious effects of radiation, few studies have actually systematically focused a possible role of low-dose brain radiation to beneficially interfere with the pathological processes associated with neurodegenerative disorders such as Alzheimer’s disease (AD)—a disorder associated with pathological accumulation of intracellular (pTau-neurofibrillary tangles) and extracellular (*β-*amyloid neuritic plaques) misfolded proteins across specific vulnerable regions of the brain^43^. Interestingly, a recent study in transgenic rodents described a reduction of *β*-amyloid plaque loads in radiation-treated animals^44^. However, no pTau or APP expression level changes across different brain regions have ever been described after a single low-dose total-body radiation using a large normal mammalian brain.

We have focused on the identification of some possible protein expression level changes and neuropathological consequences due to a single exposure of low-dose total-body radiation after a relatively short period of time (4 weeks) in a large mammalian brain (swine), and we found:

1. Lower levels of pTau (CP13) in the FC and H region of RAD-versus SH-animals;
2. Lower levels of APP and GAP43 in the CRB of RAD-versus SH-animals;
3. Higher level of GFAP in the H of RAD-versus SH-animals;
4. Higher level of DNA-polymerase-*β* in the FC of RAD-versus SH-animals.

No significant changes of microglial cells reaction (as detected by either soluble IBA-1 levels or immunohistochemistry/morphological changes) or loss of myelin (as detected by levels of soluble MBP or through LFB stain assessment) were found. Importantly, these findings on early molecular changes in a normal large mammalian brain in the absence of obvious neurohistological changes or macroscopic brain lesions support the hypothesis that early specific molecular changes do occur and are detectable at 4 weeks post-radiation across different regions of the brain. Remarkably, the lower levels of pTau, APP and GAP43 observed across the different examined regions of the normal swine brain were not associated with any obvious histological or morphological change, sign of microglial activation or demyelination phenomena, which are processes more frequently observed at a later stage during the progression of the post-radiation process especially following higher cumulative doses (> 6 months/years post-radiation)^45^. Moreover, our findings show that the neuroanatomical regions involved in this *radiation-induced protein level reduction* are not uniformly altered along the same trajectory across the different regions of the brain analyzed in this study. Rather, our findings show that specific molecular changes can involve different brain regions at different degrees and rates at a specific time point after radiation (4 weeks in our experiment). This differential post-radiation timing may be due to the intrinsic radiosensitivity to *γ*-rays in each specific cerebral region linked to their genetically-determined intrinsic metabolic rate and function as well as to the total radiation dose administered^46–48^. These differential radiosensitivity-related effects across different brain regions and their possible controlled modulation could actually lead to more precise and effective protocols selectively tuned for a specific region of the brain, or a group of disease-related neuroanatomical regions, in the context of a particular brain disease and related radio- vulnerability (or radio-sensitivity).

The reduction of pTau levels induced by total-body radiation was observed in the absence of corresponding significant changes of all-Tau forms (HT7) across all the examined regions. The unchanged levels of total-Tau protein were also indirectly confirmed by the unchanged levels of *β*-tubulin (Fig. 2), a protein strictly associated with Tau for the stabilization of microtubules^49^. It appears that one of the early effects of the low-dose radiation on intracellular Tau levels is the change of the ratios between phosphorylated and dephosphorylated forms. Intriguingly, the observed phenomenon of decreased pTau levels induced by low-dose radiation contrasts with the opposite phenomenon observed in AD pathogenesis (and other tauopathies) where a progressive process of Tau hyperphosphorylation is instead associated with increased levels of insoluble pTau and its consequent accumulation in neurons and astroglial cells across the cerebral cortex ^50,51^. Similarly, although through different biochemical mechanisms, the observed decrease of APP levels could also represent another pathway through which low-dose brain radiation could be employed as a tool to reduce the biochemical substrates of *β*-amyloid plaques and consequently reduce their related toxic effects^52^.

Importantly, these new findings need to be confirmed in terms of safety and long-term efficacy by using different types of animal experiments and following clinical trials in humans. At the time of this investigation, a total of 13 human clinical trials employing low-dose radiation in the context of dementia, and more specifically AD, have been completed or are currently ongoing. For specific information about each trial see Supplementary Fig. 31 and https://clinicaltrials.gov.

Intriguingly, the hyperphosphorylation process of Tau (as measured by CP13 levels) is a process normally present during brain development, and it is one of the earliest events that occurs during the pathogenesis of different neurodegenerative disorders^53^. Tau hyperphosphorylation is hypothesized to be a complex molecular mechanism whereby a single and then multiple amino acid sites across the entire amino acid sequence of the protein are consecutively and progressively hyperphosphorylated leading to abnormally high levels of insoluble pTau followed by its intracellular pathological accumulation ultimately affecting normal neuronal and non-neuronal (glia) cellular functions^50,51^. Our findings show that at least three of the possible enzymes normally activated during Tau phosphorylation/dephosphorylation processes (GSK3*β*, pGSK3*β* [Y216], PP2A-*βα*) remained unchanged after 4 weeks from the radiation in the brain regions examined in this study. The only exception was represented by a reduction of GSK3*β* in the CRB. These data suggest that both kinase and phosphatase enzymes could have reached a steady-state equilibrium in those examined brain regions at that specific post-radiation time point (4 weeks). Nonetheless, we cannot exclude that other phosphorylation/dephosphorylation enzymes or biochemical mechanisms might be involved in the interactions between pTau and the effects of radiation or that other chemical reactions (e.g. methylation) may occur.

Furthermore, our data show that lower levels of Ser202-phosphorylated-Tau (as detected by CP13 levels) in the FC and H regions were accompanied by concomitant lower levels of APP and GAP43 in the CRB. Intriguingly, a series of studies described close and complex interactions between APP and GAP43 in different regions of the rodent brain^54,55^. APP is the precursor protein for 1–40 *β*-amyloid protein, one of the two proteins (pTau being the other), that pathologically accumulates (in the form of extracellular 1–42 *β*-neuritic plaques) in subjects diagnosed with AD and it has been shown to interact with GAP43 in mechanisms of axonal generation and neuroplasticity as well^56^. On the other hand, GAP43 is a protein known to be involved in multiple structural and functional aspects of axonal formation during neosynaptogenic processes (during developmental period) and reparative neuronal mechanisms (during adult life)^57^. In our study, a decreased level of APP and GAP43 was unexpected, but it was even more surprising to observe a post-radiation effect only in the CRB. This latter finding may be related to the well-established notion that the CRB is one of the most resistant regions of the CNS to AD pathology in comparison to other regions of the brain (H or FC, for example). Remarkably, this AD-pathology resistance is maintained even during the late stages of the natural progression of AD. The notion about the relative resistance of the CRB against the pathological accumulation of pTau and *β*-amyloid lesions has been attributed to some genetic or biological protective factor, which remains mostly unknown. If there are direct interactions between APP and GAP43 in the CRB and why this particular region of the CNS appears to be more susceptible to a low-dose of brain radiation compared to other brain regions such as H or FC remains elusive, and it represents a fascinating question deserving of future research efforts.

In general, the reduction of soluble levels of pTau, APP, and GAP43 across different mammalian brain regions could also be due to the terminal effects of more basic mechanisms associated with DNA (or RNA) damage or repair activities, especially in those regions of the brain more susceptible to radiation. In support of an early DNA radiation-induced activation, we did find an increased level of DNA-polymerase-*β* in the FC region. This is not so surprising since the FC is one of the latest neuroanatomical regions to ontogenetically and phylogenetically fully develop during the CNS maturation process as related to its high level of circuital complexity and biological instability^58^. Importantly, though, DNA-polymerase-*β* is one of the enzymes involved in reparative processes and in various duplicative and reparative DNA mechanisms that could have been indeed triggered by low-dose brain radiation.

In contrast to the lower levels of pTau, APP, and GAP43 in FC, H and CRB regions, but consistent with previous clinical and experimental observations, the level of GFAP in the H was higher in RAD-versus SH-animals. Furthermore, the increased level of GFAP in the H (measured by WB quantifications) was confirmed by an initial astroglial cell reaction as detected by immunohistochemistry. More specifically, based on the immunohistochemistry-microscopic inspection, the astrogliosis seems to be localized at level of the peri-dental gyrus (peri-DG) area of the H (Fig. 6)—one of the hippocampal subregions more often activated during various physiological and pathological processes affecting the H. WB and neuropathological findings suggest then the co-presence of an early neuroinflammatory response in the H associated with a reduction of other proteins levels (pTau) in the H and FC regions. Furthermore, levels of IBA-1 (a marker of microglia) and MBP (a marker of myelin), did not differ in RAD-versus SH-animals in any of the considered brain regions and no changes were observed at histological level or immunohistochemistry at high-magnification (40X) light microscopy inspection. These last findings suggest that microglial cells and oligodendrocytes may possibly react at a later stage or at a much slower rate compared to other possible more “radio-sensitive” cell types.

In general, the WB results obtained from low-dose radiation did not parallel any obvious brain lesion observable by histology stains (HE, LFB, CV) or immunohistochemistry, except for the increase of GFAP as an indication of astrogliosis in the H (Fig. 6). We would like to emphasize that increased levels of GFAP are not necessarily linked to detrimental effects since the astroglial cells are also involved in various regenerative and neurogenesis phenomena^59^. Moreover, the absence of immunohistochemistry-based histopathological brain lesions (e.g. intracellular inclusions) in RAD-versus SH-animals indirectly confirmed the reduced levels of pTau, APP, and GAP43 across all the examined regions. In fact, those reduced levels of soluble proteins could have prevented the generation of the biochemical conditions necessary to determine molecular changes (e.g. increase levels of Tau, pTau/Tau ratio abnormalities) that are expected to induce the pathological accumulation of insoluble hyperphosphorylated-Tau in long-distance cortical neurons for example.

One major strength of this study is that we have used a large mammalian brain under normal anatomical and physiological conditions in order to observe possible molecular and neurohistopathological changes due to low-dose brain radiation after 4 weeks. These results in swine are relevant since the swine brain represents a neural system that is more similar to humans than rodents in terms of neuroanatomy, neurocircuitry complexity, cerebro-vascular physiology, liver metabolism, pharmacology, etc. This makes swine brain an excellent model for brain radiation research while searching for its potential beneficial effects at molecular and behavioral level. These species-related considerations are especially important when considering that recent studies have shown that the gene responses to radiation (e.g. in the blood) vary greatly across different species^60^.

To the best of our knowledge, these molecular and neuropathological findings observed 4 weeks following single low-dose total-body radiation in a large mammalian brain are unique and of special relevance for possible future therapeutic applications to conditions affecting the CNS, especially those conditions associated with different types and mechanisms of misfolded protein accumulation. However, future larger studies are necessary to precisely define other possible important brain-radiation effects and possible interacting or modifying factors, the minimal necessary effective dose to administer, best fractionated scheme for each specific brain region and condition, the definition of possible stereotactic approaches, the genetic background-based response, the age-related effects and other possible factors not determined yet. Establishing all these parameters could greatly improve the beneficial and therapeutic applicability of low-dose brain radiation during the early phases of specific neurodegenerative conditions in humans. If confirmed at a larger scale, these new experimental data could have a major clinical and societal impact for the use of low-dose brain radiation as a new additional neuro-radiotherapeutic tool for neurodegenerative disorders, including but not limited to, tauopathies such as AD, progressive supranuclear palsy (PSP) and chronic traumatic encephalopathy (CTE).

## Methods

### Animals

All animal handling procedures were in compliance with guidelines from the National Research Council for the ethical handling of laboratory animals and approved by the Uniformed Services University (USU) and Armed Forces Radiobiology Research Institute (AFRRI) Institutional Animal Care and Use Committees (Protocol PHA-18-942) as well in compliance with the ARRIVE guidelines (https://arriveguidelines.org/arrive-guidelines). Male Gottingen minipigs ranging in age from ~ 6.0 to 6.5 months were purchased from Marshall Farms Group Ltd. USA (North Rose, NY, USA). All swine were kept in a barrier facility for animals accredited by the Association for Assessment and Accreditation of Laboratory Animal Care International. Swine were housed in pairs. Animal rooms were maintained at 21 ± 2 °C, 50 ± 10% humidity, and 12-h light/dark cycle with food and water available ad libitum. Swine were acclimatized to the animal facility for 3 days prior to the start of the study. Swine were divided into the following groups (total animal number was achieved over the course of 4 separate experiments):

1. Sham + Vehicle (*n* = 4)
2. Radiation + Vehicle (*n* = 6)
3. Sham + Captopril (*n* = 6)
4. Radiation + Captopril (*n* = 6)

Approximately two weeks after arrival, swine receiving total-body radiation were deeply anesthetized with Ketamine/Xylazine (4.4 mg/kg–2 mg/kg) and transported to the High Level Cobalt facility at AFRRI. While under deep anesthesia, swine were positioned in supportive slings and exposed one at a time, bilaterally, to a target total body dose of 1.79 Gy of Cobalt (^60^Co) radiation delivered at a dose rate of 0.485–0.502 Gy/min as previously described ^35^. After radiation procedures, each animal was transported back to the animal facility for recovery. Swine assigned to the sham (SH) groups were also deeply anesthetized with Ketamine/Xylazine (4.4 mg/kg–2 mg/kg) in the animal facility but were not transported to the Cobalt facility. Treatment with either captopril (USP Grade C8856, Sigma-Aldrich, St Louis, MO, USA), dissolved in sterile water and mixed in yogurt) (10 mg/animal, PO, twice daily) or Vehicle (yogurt, PO, twice daily) began at 4 h post-radiation or control equivalent and continued twice daily for 12–14 days.

## Tissue collection

Euthanasia was performed with an intracardial injection of Euthasol (4.5 ml/kg) and confirmed by lack of heartbeat. Each animal underwent necropsy procedures for the sampling of different organs (spleen, lung, liver, kidney, jejunum, skin, bone marrow—to be used by collaborators) as well as the collection of the entire brain and spinal cord.

Each brain was grossly inspected and longitudinally dissected across the median line of the corpus callosum to separate the two cerebral and cerebellar hemispheres. The left hemisphere of each animal was quick-frozen in chilled liquid isopentane on dry ice (destined for molecular analyses). Frozen brains were kept at − 80 °C until use. The right hemispheres were placed in 10% buffered formalin for tissue fixation (for histological and immunohistochemistry purposes).

### Protein extraction and western blot (WB) procedures

Frozen brains were warmed to − 20 °C in a cryostat. Each left cerebral hemisphere was cut into 100 µm thick sections and further microdissected into three main anatomical regions: frontal cortex (FC), hippocampus (H), and cerebellum (CRB). The dissections were guided by following the Gottingen Minipig Brain Atlas (https://www.cense.dk/miniswine_atlas).

Samples containing both gray (GM) and subjacent white matter (WM) from all animals and from each dissected neuroanatomical region (FC, H, CRB) were homogenized in glass dounce homogenizers with ice cold lysis buffer (1 ml/100 mg tissue), which contained the following: 50 mM Tris–HCl (pH 8), 1% Igepal, 150 mM NaCl, 1 mM EDTA, 1 mM PMSF, 1 mM NaF, 1:100 protease inhibitor cocktail (Sigma-Aldrich, P2714, St. Louis, MO, USA). All samples were centrifuged at 12,000 × *g* for 20 min and supernatants collected, aliquoted and frozen at – 80 °C. Total protein content from each brain region (FC, H, CRB) was determined using the Micro BCA assay (Thermo-Fisher Scientific, 23235, Waltham, MA, USA). 20 µg of protein per sample, for all brain regions listed, were loaded on Novex Nupage 4–12% Bis–Tris Gels (Life Technologies, NP0329, Carlsbad, CA, USA) and were electrophoresed at 200 V constant for 30 min. Gels were transferred to PVDF membranes using the iBlot2 dry transfer method (Life Technologies, IB21001, Carlsbad, CA, USA). Membranes were blocked in 5% milk in 1× TBST for 1 h at room temperature (RT). The primary antibodies (see paragraph below) were diluted to the appropriate working concentrations in 5% milk in 1× TBST and incubated on the membranes overnight at 4 °C. Membranes were then rinsed 3 × 5 min in TBST. Appropriate HRP tagged secondary antibodies (see paragraph below) were diluted 1:2000 in 5% milk in 1× TBST and incubated on the membranes for 1 h at RT. Membranes were rinsed 3 × 5 min in TBST and 1  × 5 min in TBS. Membranes were incubated with chemiluminescent substrate (SuperSignal West Pico Chemiluminescent Substrate, Thermo-Fisher Scientific, 34577, Waltham, MA, USA) for 1 min and imaged on the LiCor C-Digit Blot Scanner (LiCor Biosciences, Lincoln, NE, USA). All membranes were stripped one time with Restore Plus Stripping Buffer (Thermo-Fisher Scientific, 46430, Waltham, MA, USA), for 10 min, rinsed with TBS and processed for immunoblotting as described above using GAPDH (1:40,000, Millipore-Sigma, AB2302, Billerica, MA, USA) for the loading control. Densitometry was performed with NIH ImageJ software (2.0.0) with all protein signal intensities normalized to GAPDH signal intensity.

### Primary antibodies

To examine possible neuronal, astroglial, and microglial protein expression level changes occurring across the three selected brain regions (FC, H, CRB) in radiated (RAD)-versus sham (SH)-animals four weeks after the radiation time-point, the following primary antibodies were used: CP13 (which recognizes Tau protein phosphorylated at amino acid serine in position 202 [Ser-202])^61^ (1:250; this antibody was a gift of Professor Peter Davies, Albert Einstein College of Medicine, Bronx, NY, USA); HT7 (which recognizes all forms of Tau protein) (1:500; Thermo-Fisher Scientific, MN1000, Waltham, MA, USA); and an anti-*β*-tubulin antibody (1:5000; Thermo-Fisher Scientific, 322600, Waltham, MA, USA). Levels of HT7 and anti-*β*-tubulin antibody were measured to determine expression level changes of all Tau forms and possible corresponding associated changes in *β*-tubulin levels due to Tau interaction with tubulin molecules to stabilize microtubules^49^. In addition, we measured the expression levels of some kinase and phosphatase enzymes normally associated with the phosphorylation status of Tau by measuring the following: GSK3*β* (1:500; BD Biosciences, 610201, San Jose, CA, USA), a constitutively active kinase responsible for phosphorylation of Tau^62^; pGSK3*β* (Y216) (1:500; BD Biosciences, 612312, San Jose, CA, USA), a phospho-activated [Y216] kinase related to Tau^63,64^; and PP2A-*βα* (1:500; Millipore-Sigma, cat.#05-592, Billerica, MA, USA), a primary Tau phosphatase^65,66^. We also explored possible DNA damage/repair level changes by measuring DNA polymerase-*β*^67^ (1:1000; cat. #ab26343, Abcam, Cambridge, MA, USA).

We hypothesized that synapse-associated proteins could be altered in RAD-versus SH-animals, so we also measured the expression levels of GAP43 (1:5000; cat.#ab232772, Abcam, Cambridge, MA, USA)—a marker associated with neosynaptogenesis, neuroplasticity, and axonal regeneration^68^. Furthermore, we measured expression levels of proteins known to be associated with longer term post-radiation effects (e.g. neuroinflammation, microglia activation, and demyelination) by measuring levels of glial fibrillary acidic protein (GFAP) (1:10,000; Leica Biosystems, cat.#NCL-L-GFAP-GA5, Newcastle Upon Tyne, UK), a marker of astroglial cells^69^, ionized calcium-binding adapter molecule 1 (IBA-1) (1:1000; cat.#ab178847, Abcam, Cambridge, MA, USA), a marker for microglial cells^70^, and myelin basic protein (MBP) (1:2000; cat.#ab7349, Abcam, Cambridge, MA, USA), a marker of myelination^71^. Lastly, we measured levels of amyloid precursor protein (APP) (1:1000; Millipore-Sigma, cat. #MAB348, Billerica, MA, USA), a marker associated with diffuse axonal injury (DAI) and involved in the pathogenesis of Alzheimer’s disease (AD)^72–75^.

### Secondary antibodies

The following HRP tagged secondary antibodies were used: Goat anti-mouse (1:2000; cat.#ab97040, Abcam, Cambridge, MA, USA), Goat anti-rabbit (1:2000; cat.#ab97080, Abcam, Cambridge, MA, USA), Goat anti-rat (1:2000; cat.#ab97057, Abcam, Cambridge, MA, USA), and Rabbit anti-chicken (1:5000; cat.#AP162P, Millipore-Sigma, Billerica, MA, USA).

### Neurohistological and immunohistochemistry procedures

Tissue blocks from each animal were uniformly processed using an automated tissue processor (ASP 6025, Leica Biosystems, Nussloch, Germany). After tissue processing, each tissue block was embedded in paraffin and cut in a series of 20 5 µm-thick consecutive sections. The first three sections were respectively selected for hematoxylin and eosin (H&E), Luxol fast blue (LFB) and Cresyl Violet (CV) stains, while the remaining sections were available for immunohistochemistry procedures. Immunohistochemistry procedures for each antibody on all dissected brain regions were performed using a Leica Bond III automated immunostainer with a diaminobenzidine chromogen detection system (DS9800, Leica Biosystems, Buffalo Grove, IL). The following antibodies were used: anti-phosphorylated Tau (CP13)^61^ (mouse antihuman monoclonal antibody, 1:2000, epitope retrieval time 10 min; this antibody was kindly donated by Dr. Peter Davies, Albert Einstein College of Medicine, New York City, NY); anti-all forms of Tau (HT7) (mouse antihuman monoclonal antibody, 1:150, epitope retrieval time 10 min, MN1000, ThermoScientific, Waltham, MA, USA); anti-amyloid precursor protein (APP)^72–75^ (mouse antihuman monoclonal antibody clone 22c11, 1:10, epitope retrieval time 10 min, MAB348, EMD Millipore, Burlington, VT, USA); anti-glial fibrillary acidic protein (GFAP)^69^ (mouse antihuman monoclonal antibody GA5, 1:250, with bond heat-induced epitope retrieval, epitope retrieval time 10 min, PA0026, Leica Biosystems, Wetzlar, Germany); anti-ionized calcium-binding adapter molecule 1 (IBA-1)^70^ (rabbit polyclonal, 1:100, epitope retrieval time 10 min, Wako 016-20001, FUJIFILM Wako Pure Chemical Corporation, Osaka, Japan); and anti-myelin basic protein (MBP)^71^ (mouse monoclonal antibody, 1:100, MBP101, ab62631, Abcam, Cambridge, MA, USA).

All stained sections were scanned by an Aperio scanner system (Aperio AT2—High Volume, Digital whole slide scanning scanner, Leica Biosystems, Inc., Richmond, IL) and stored in Biolucida system, a hub for 2D and 3D image data (version 2017, MBF Bioscience, Williston, VT, USA) for further assessment and analyses to verify the immunoreactivity (IR) for each antibody and histological distribution of possible lesions and their severity across all examined brain regions and conditions. A preliminary neuropathologic assessment for each section, region and animal was performed using Aperio ImageScope (Aperio ImageScope, version 2016, Leica Biosystems, Inc.). Compared to WB analyses, we examined more neuroanatomical regions for the immunohistochemistry evaluation: frontal cortex (FC), parietal and temporal cortex (PTC), basal ganglia (BG), hippocampus (H), occipital cortex (OC), cerebellum (CRB), and brainstem (BS). After a preliminary ImageScope inspection (max 20X), a Zeiss Imager A2 (ImagerA2 microscope, Zeiss, Munich, Germany) bright-field microscope inspection at higher magnification (40X, 63X oil-immersion objectives) was used to identify and digitally photograph possible histopathologic details as needed.

### Statistics

Initially, we considered the following four groups of animals: sham-vehicle, sham-captopril, radiation-vehicle, and radiation-captopril. For each considered protein (CP13, HT7, *β*Tubulin, APP, GAP43, GFAP, DNA polymerase-*β*, MBP, IBA-1, GSK3*β*, pGSK3*β* and PP2A-*βα*) we ran a series of one-way ANOVA analyses for each examined neuroanatomical region (FC, H, CRB). None of the ANOVA results showed differences between sham-vehicle versus sham-captopril or between radiation-vehicle versus radiation-captopril groups. See each scatterplot graph and single ANOVA results for all WB obtained data in the Supplementary Figs. 2–7. Pooling together all available swine in two main groups allowed for an increase in the total number of animals available for each group producing a larger sample size with a higher statistical power and significance. Thus, only the following two groups of animals were considered for the WB quantifications: RAD (*n* = 12) and SH (*n* = 10 for FC and H; n = 7 for CRB). Three CRB from the SH- group were used in other experiments and thus not available for these WB analyses.

Then, for each soluble protein considered in the study, a series of separate two-tailed unpaired *t*-tests was performed between RAD-versus SH-groups. Statistical significance for each measured protein expression level in each anatomical region (FC, H, CRB) examined was established when *p* ≤ 0.05.

### Ethics approval and consent to participate

All animal work was approved by the Institutional Animal Care and Use Committees at the Uniformed Services University (USU, Bethesda, MD, USA) and Armed Forces Radiobiology Research Institute (AFRRI), (Protocol PHA-18-942), in compliance with the PHS Policy on Humane Care and Use of Laboratory Animals, the NIH Guide for the Care and Use of Laboratory Animals, and all applicable Federal regulations governing the protection of animals in research.

## Supplementary information

Supplementary information

## Data Availability

The datasets used and/or analyzed during the current study and supporting the conclusions of this article are included in this article and in all supplementary materials provided. These datasets are also available from the corresponding author on reasonable request.
