# Supplementary material for: Reduction of pTau and APP levels in mammalian brain after low-dose radiation
Source: Sci Rep. 2021 Jan 26;11:2215. doi: 10.1038/s41598-021-81602-z (PMC7838187; doi:10.1038/s41598-021-81602-z)
Supplement: Supplementary file 1 — Supplementary information [file 41598_2021_81602_MOESM1_ESM.pdf]

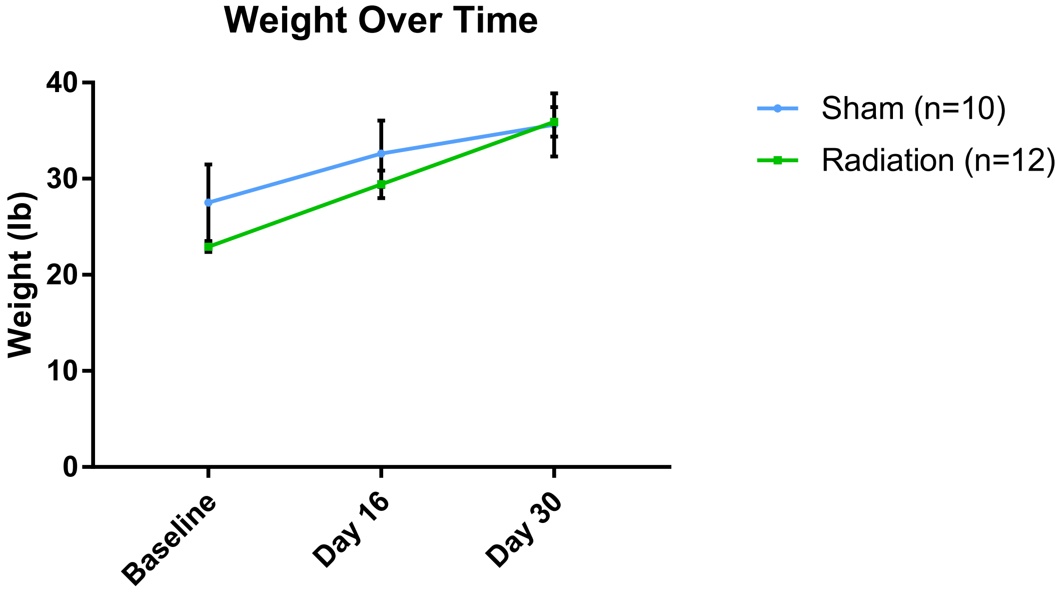

Supplementary Figure 1

a.

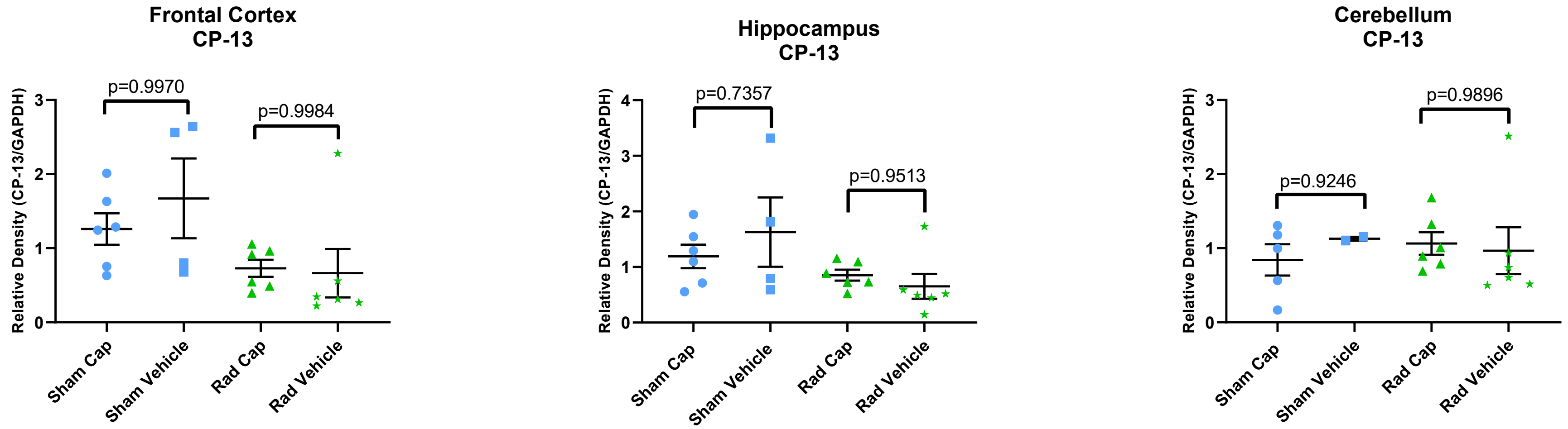

Frontal Cortex CP-13

ANOVA summary
F 2.363
P value 0.1053
P value summary ns
Significant diff. among means (P < 0.05)? No
R squared 0.2825

Hippocampus CP-13

ANOVA summary
F 2.021
P value 0.1469
P value summary ns
Significant diff. among means (P < 0.05)? No
R squared 0.2520

Cerebellum CP-13

ANOVA summary
F 0.1995
P value 0.8951
P value summary ns
Significant diff. among means (P < 0.05)? No
R squared 0.03837

b.

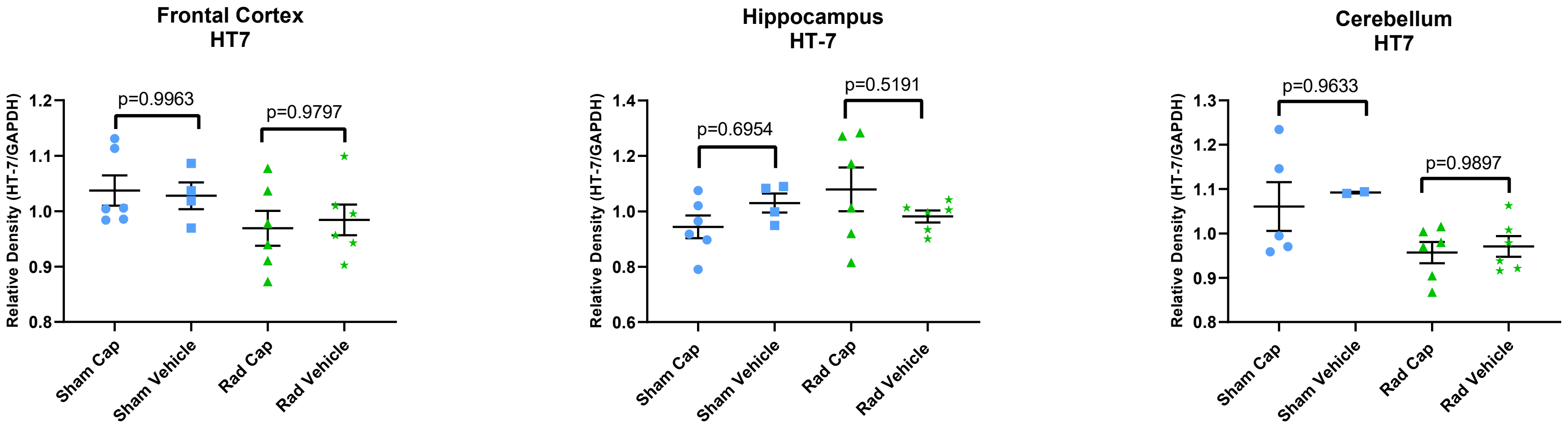

Frontal Cortex HT7

ANOVA summary
F 1.350
P value 0.2897
P value summary ns
Significant diff. among means (P < 0.05)? No
R squared 0.1837

Hippocampus HT-7

ANOVA summary
F 1.381
P value 0.2807
P value summary ns
Significant diff. among means (P < 0.05)? No
R squared 0.1871

Cerebellum HT7

ANOVA summary
F 2.745
P value 0.0797
P value summary ns
Significant diff. among means (P < 0.05)? No
R squared 0.3544

Supplementary Figure 2

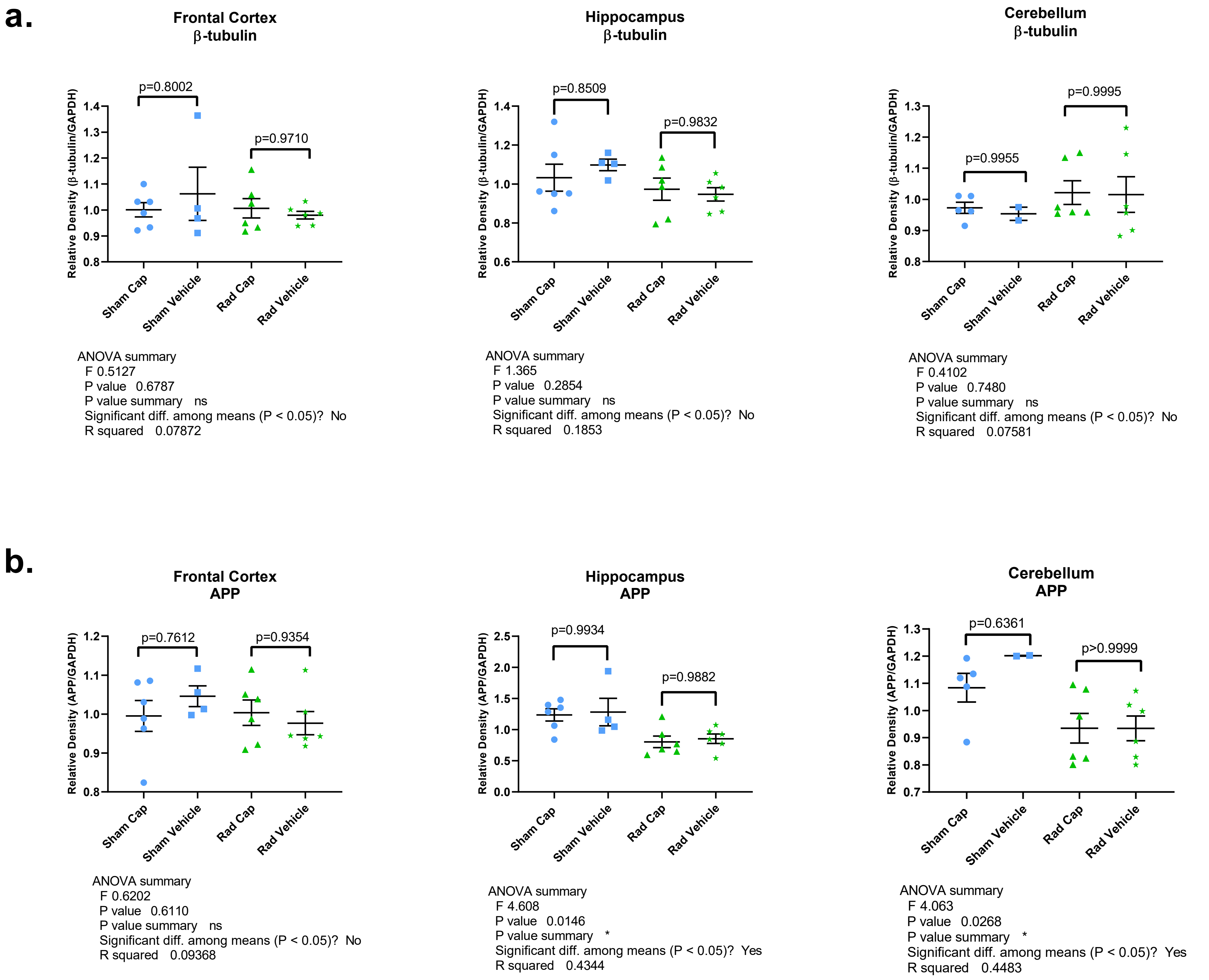

Supplementary Figure 3

a.

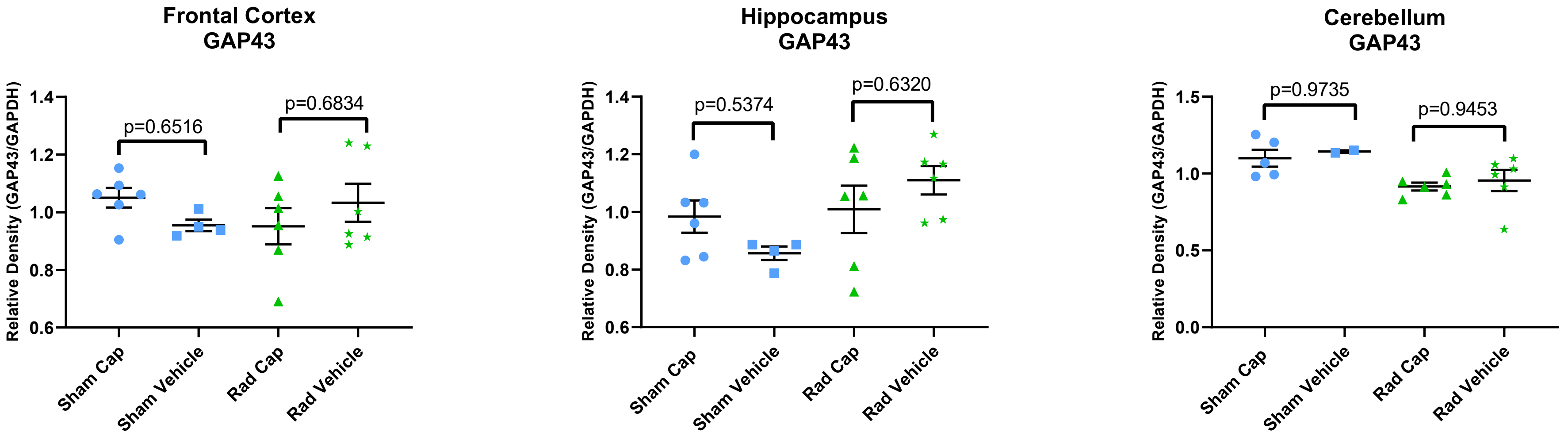

ANOVA summary
F 0.9180
P value 0.4520
P value summary ns
Significant diff. among means (P < 0.05)? No
R squared 0.1327

ANOVA summary
F 2.500
P value 0.0922
P value summary ns
Significant diff. among means (P < 0.05)? No
R squared 0.2942

ANOVA summary
F 3.295
P value 0.0497
P value summary *
Significant diff. among means (P < 0.05)? Yes
R squared 0.3972

b.

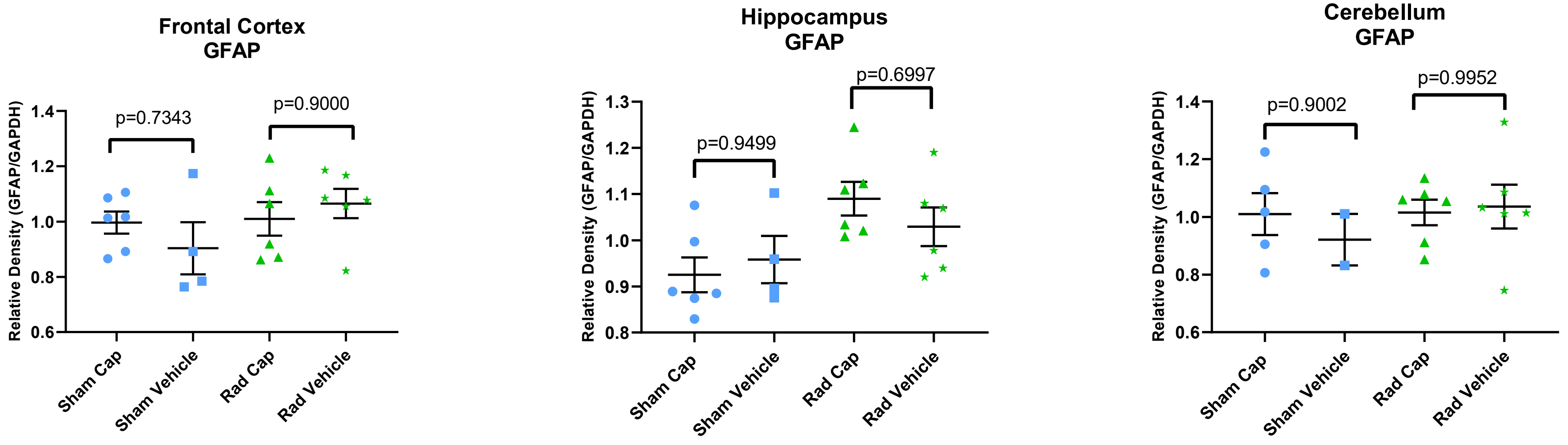

ANOVA summary
F 1.082
P value 0.3819
P value summary ns
Significant diff. among means (P < 0.05)? No
R squared 0.1528

ANOVA summary
F 3.382
P value 0.0410
P value summary *
Significant diff. among means (P < 0.05)? Yes
R squared 0.3605

ANOVA summary
F 0.2819
P value 0.8377
P value summary ns
Significant diff. among means (P < 0.05)? No
R squared 0.05337

Supplementary Figure 4

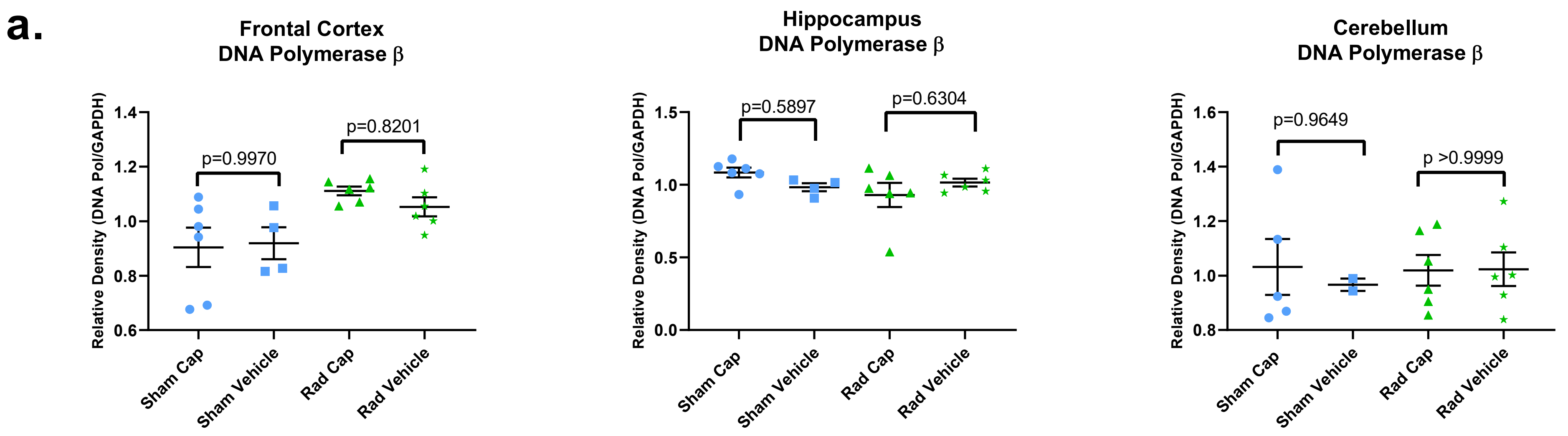

ANOVA summary
F 4.243
P value 0.0197
P value summary *
Significant diff. among means (P < 0.05)? Yes
R squared 0.4142

ANOVA summary
F 1.643
P value 0.2147
P value summary ns
Significant diff. among means (P < 0.05)? No
R squared 0.2150

ANOVA summary
F 0.07707
P value 0.9714
P value summary ns
Significant diff. among means (P < 0.05)? No
R squared 0.01518

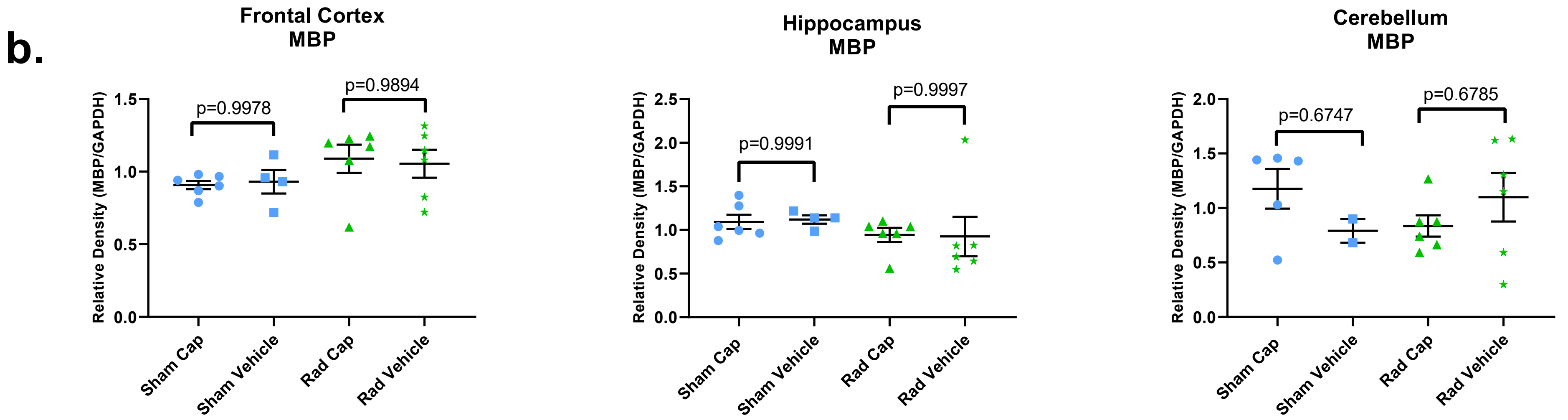

ANOVA summary
F 1.220
P value 0.3310
P value summary ns
Significant diff. among means (P < 0.05)? No
R squared 0.1690

ANOVA summary
F 0.4869
P value 0.6956
P value summary ns
Significant diff. among means (P < 0.05)? No
R squared 0.07506

ANOVA summary
F 0.9411
P value 0.4454
P value summary ns
Significant diff. among means (P < 0.05)? No
R squared 0.1584

Supplementary Figure 5

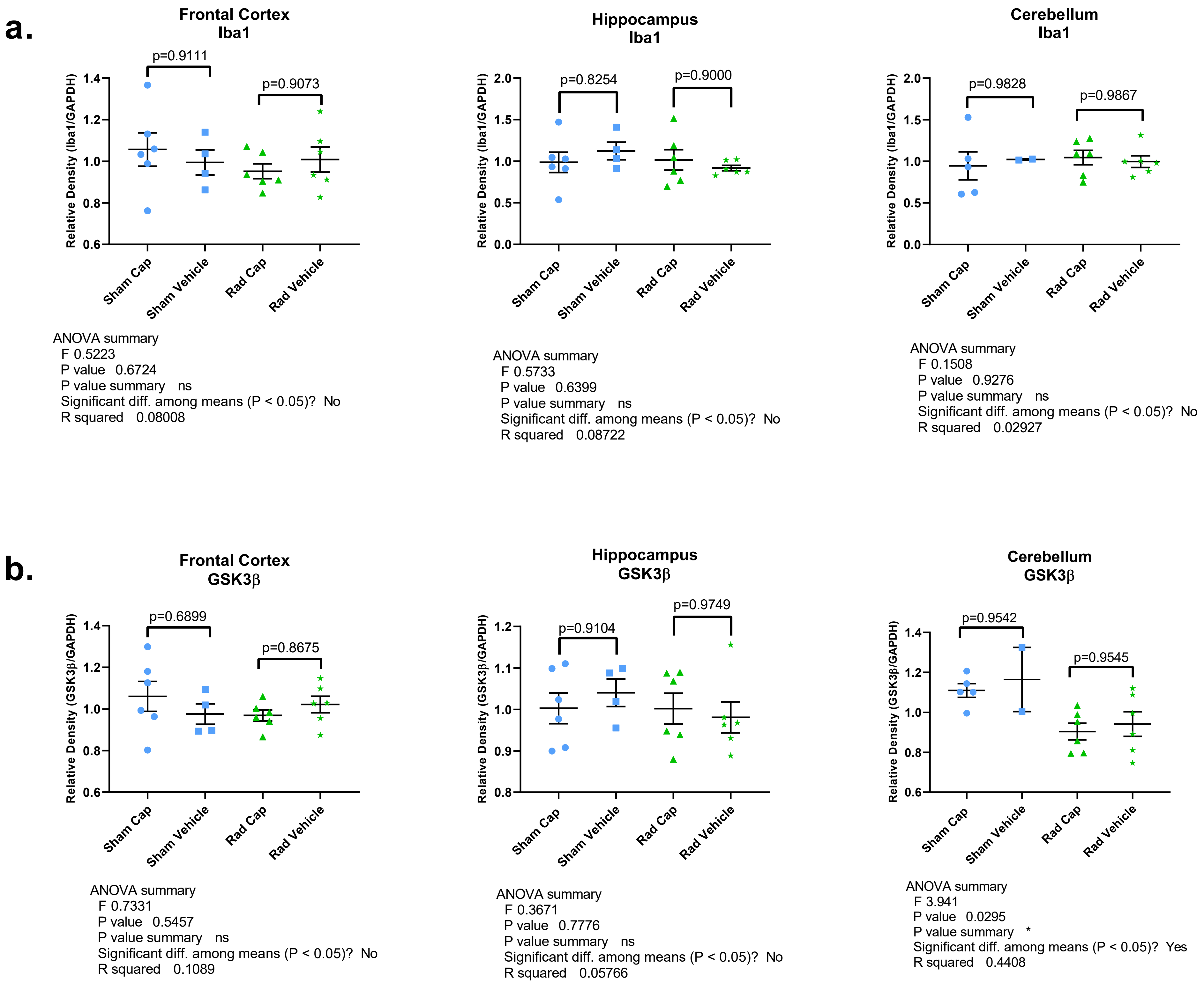

Supplementary Figure 6

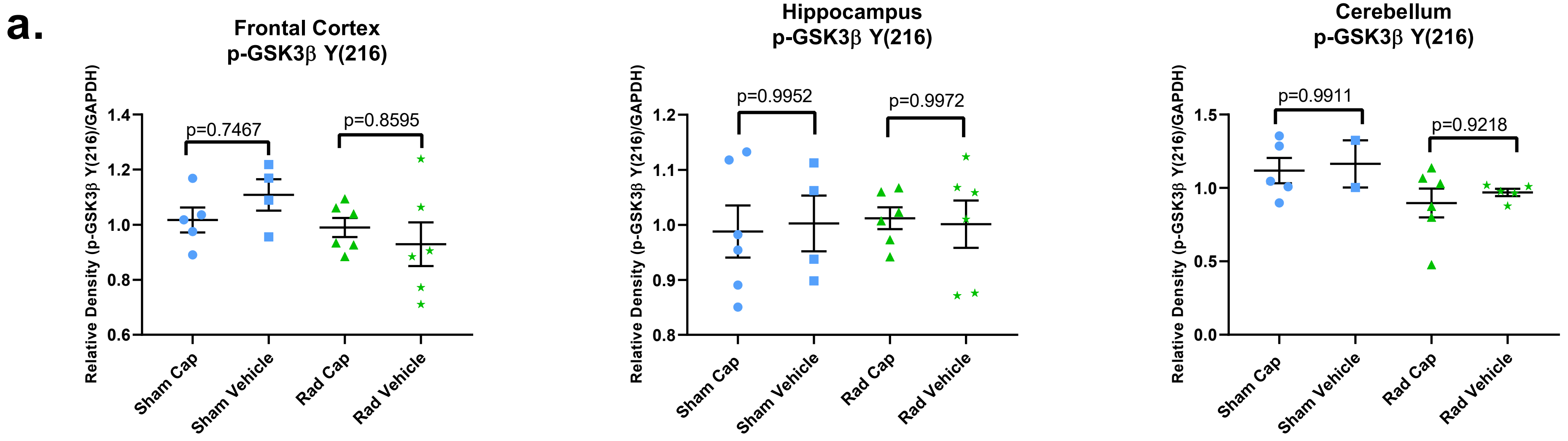

ANOVA summary
F 1.469
P value 0.2585
P value summary ns
Significant diff. among means ($P < 0.05$)? No
R squared 0.2058

ANOVA summary
F 0.06381
P value 0.9783
P value summary ns
Significant diff. among means ($P < 0.05$)? No
R squared 0.01052

ANOVA summary
F 1.764
P value 0.2001
P value summary ns
Significant diff. among means ($P < 0.05$)? No
R squared 0.2743

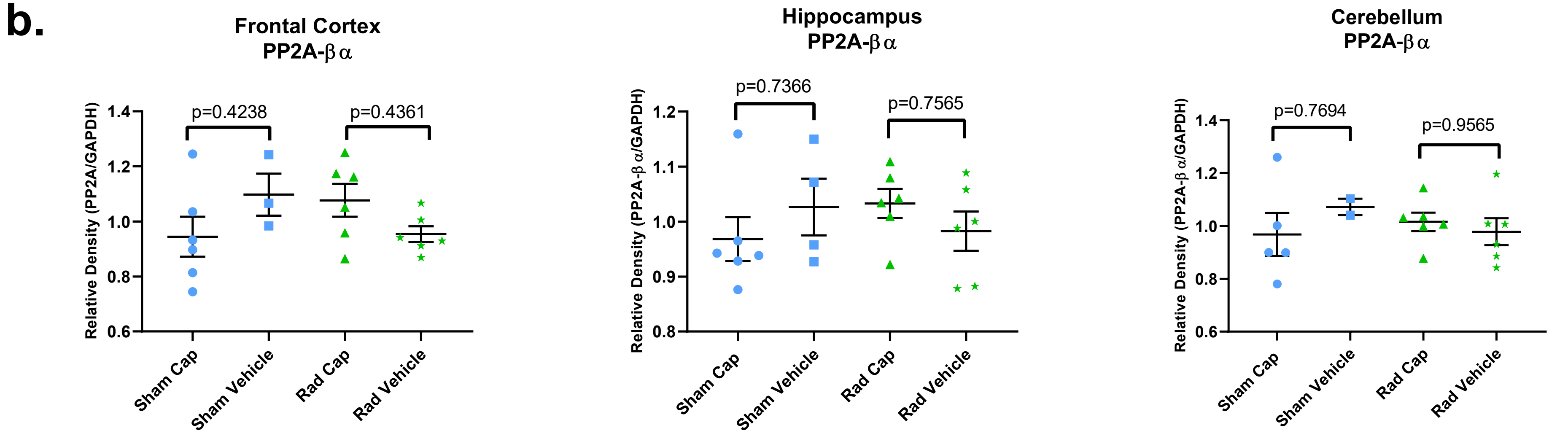

ANOVA summary
F 1.642
P value 0.2170
P value summary ns
Significant diff. among means ($P < 0.05$)? No
R squared 0.2247

ANOVA summary
F 0.7392
P value 0.5424
P value summary ns
Significant diff. among means ($P < 0.05$)? No
R squared 0.1097

ANOVA summary
F 0.3973
P value 0.7569
P value summary ns
Significant diff. among means ($P < 0.05$)? No
R squared 0.07362

Supplementary Figure 7

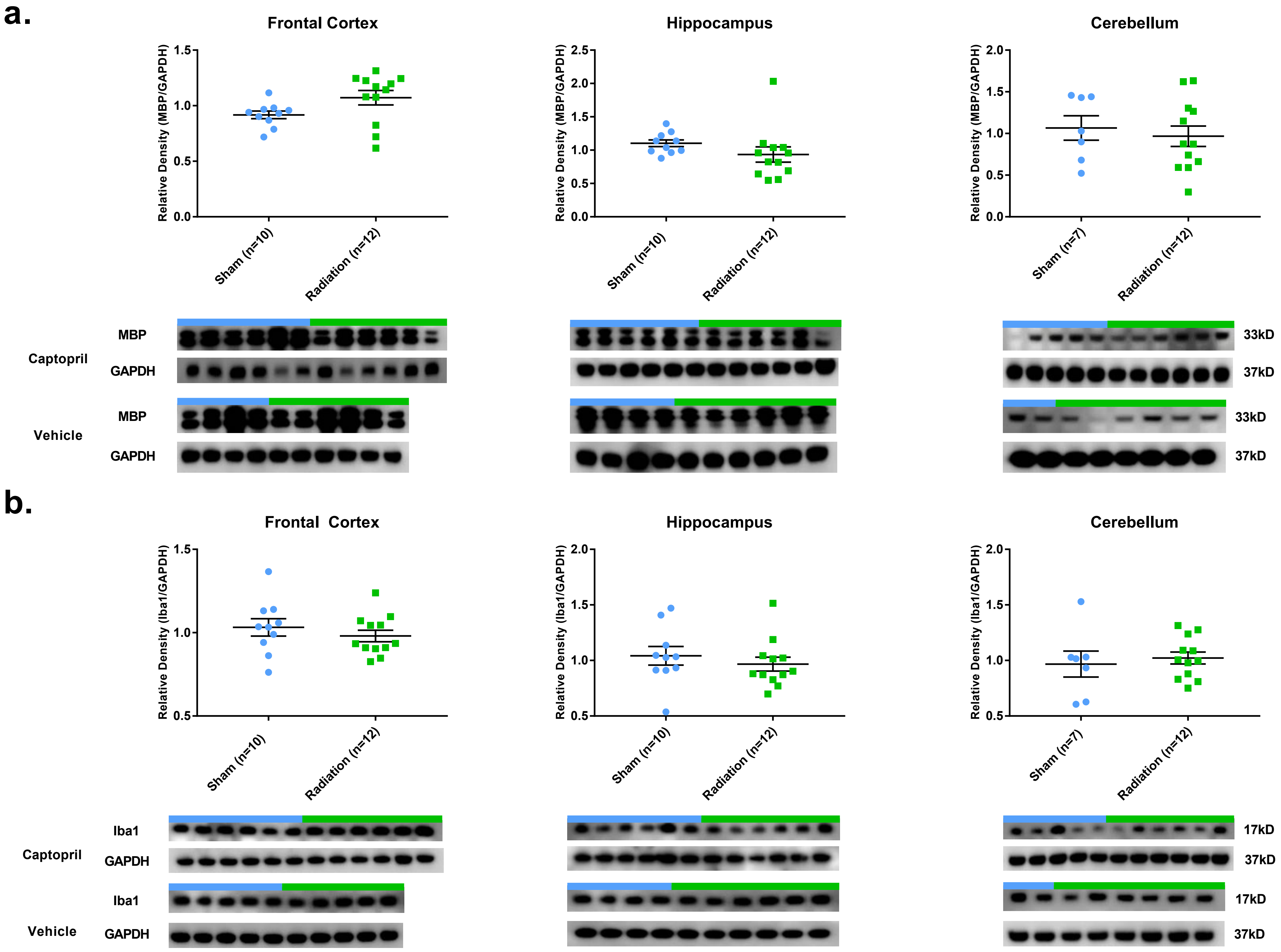

Supplementary Figure 8

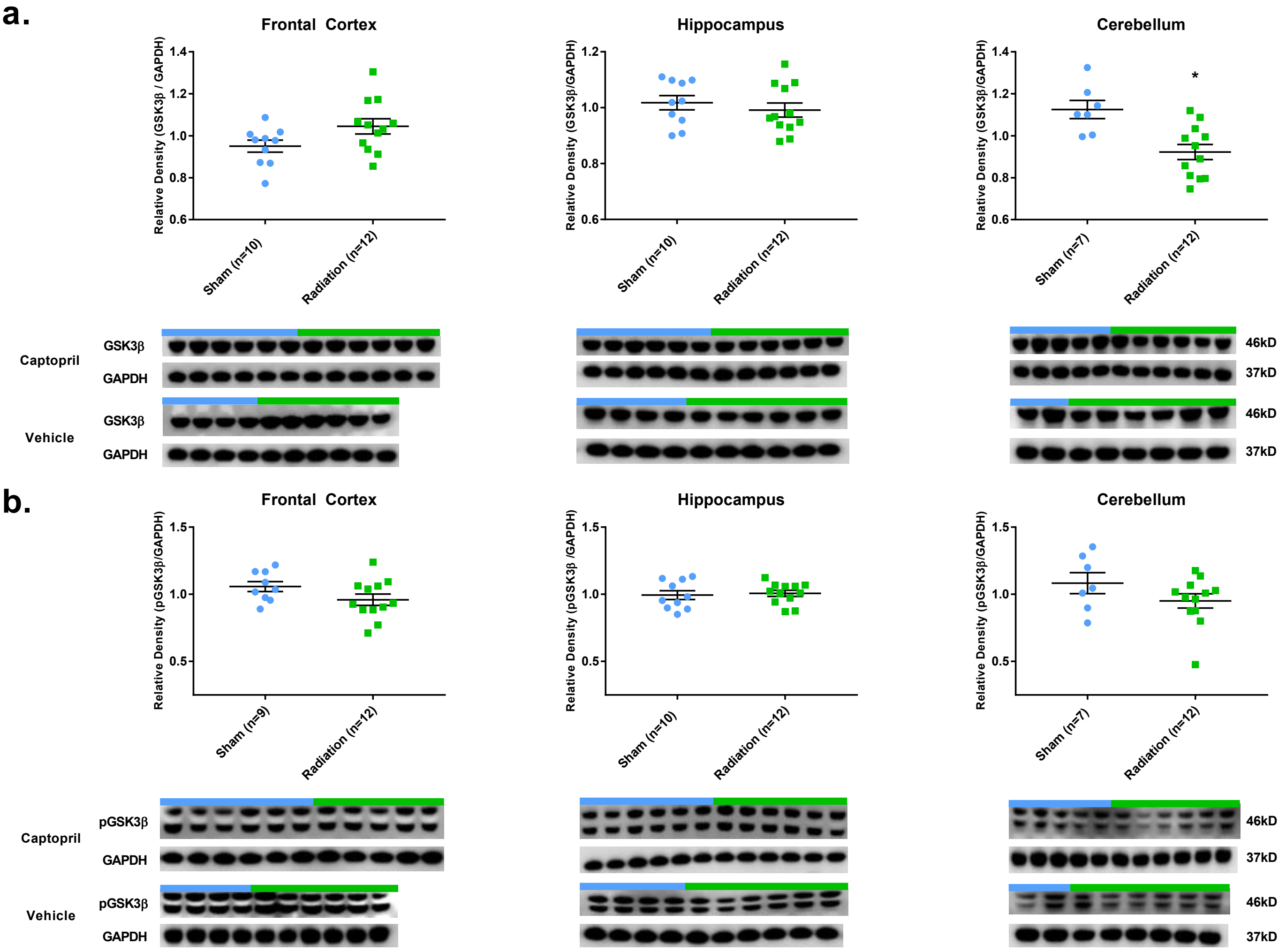

Supplementary Figure 9

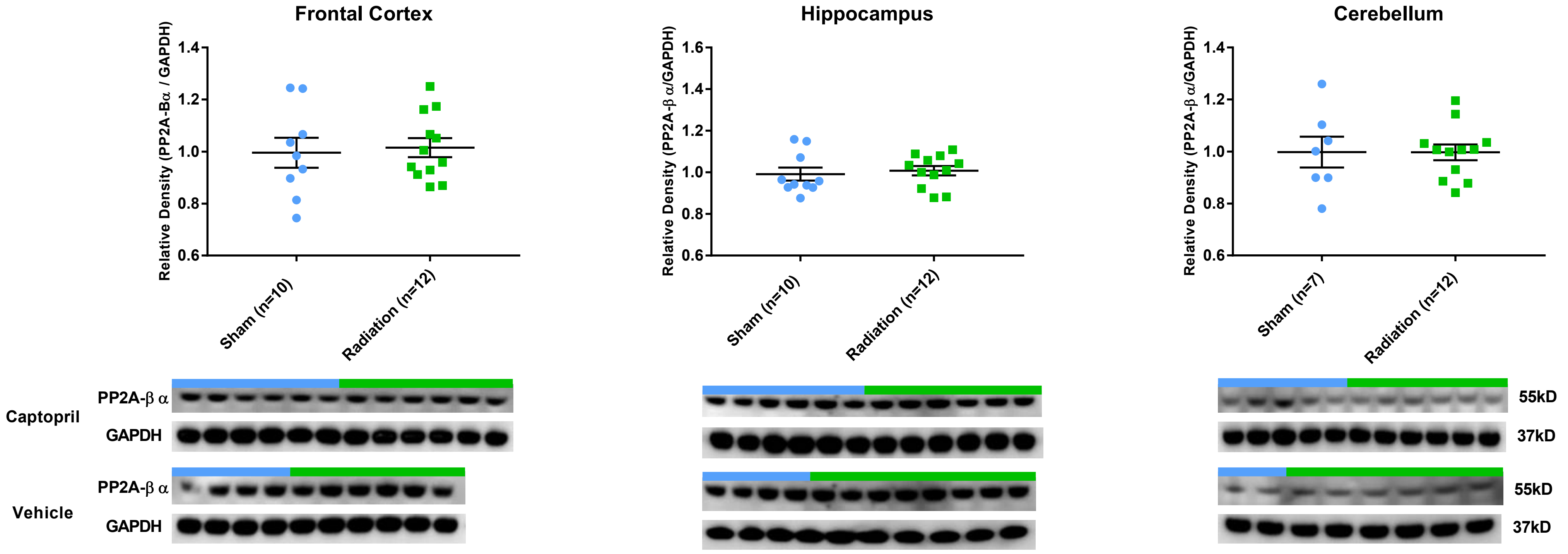

Supplementary Figure 10

CP-13 1:250
MW ~50-75 kDa

HT-7 1:500
MW ~75 kDa

β-tubulin 1:5000
MW~50 kDa

Frontal Cortex - Captopril

75kDa

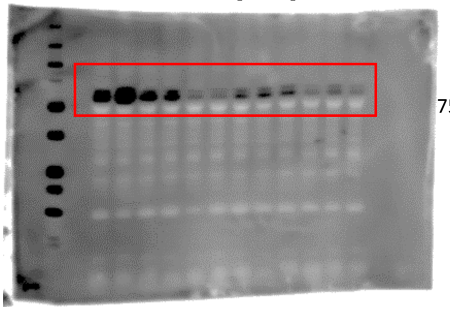

75kDa

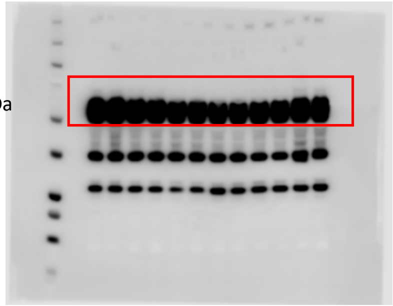

50kDa
37kDa

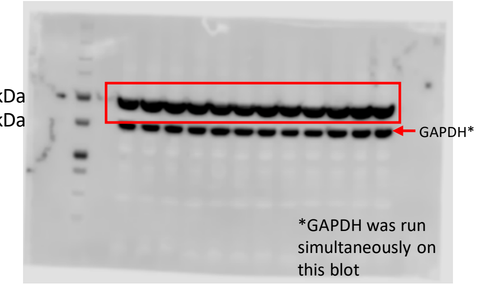

Frontal Cortex - Vehicle

75kDa

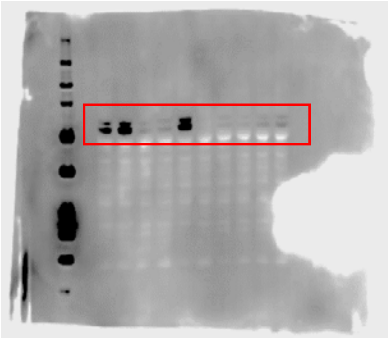

75kD.

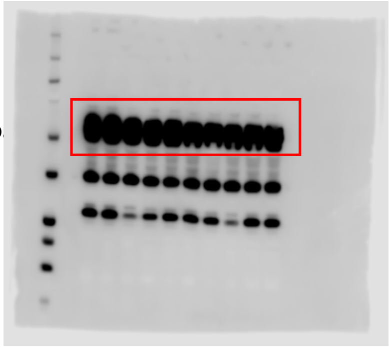

50kDa
37kDa

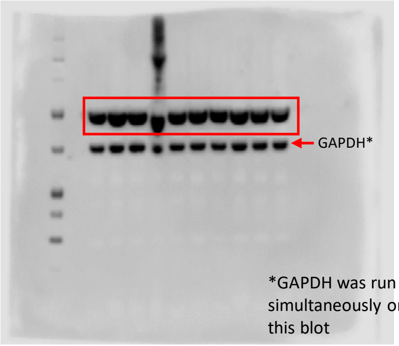

Hippocampus - Captopril

75kDa

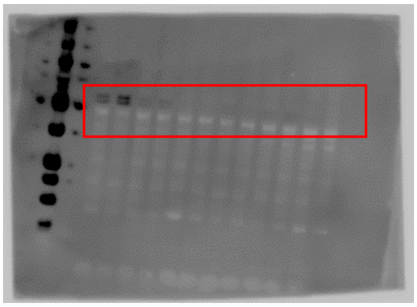

75kDa

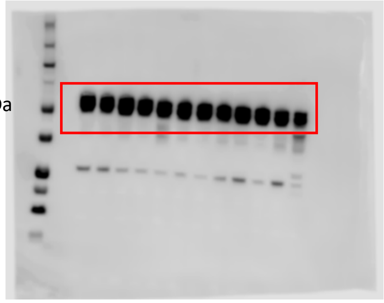

50kDa

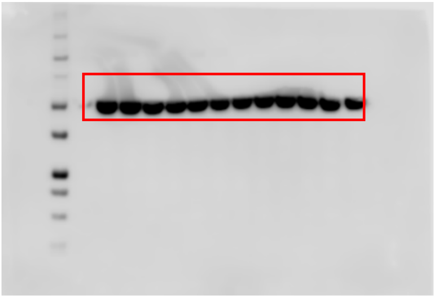

Hippocampus - Vehicle

75kDa

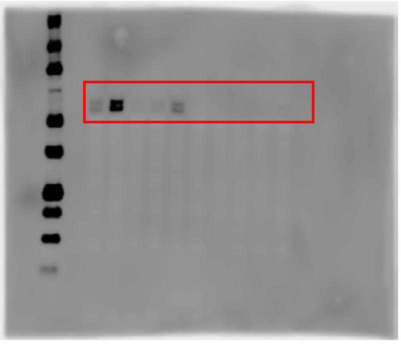

75kDa

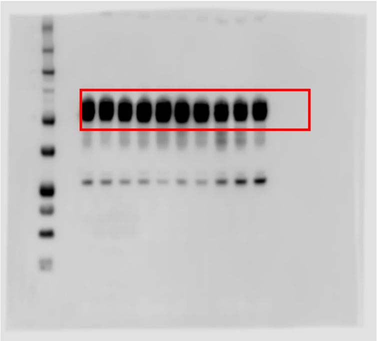

50kDa

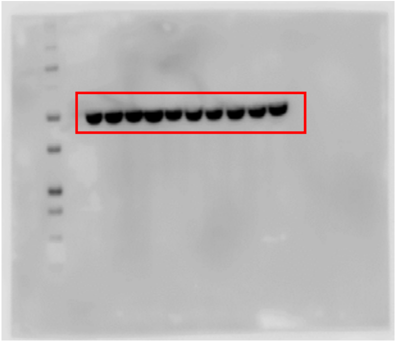

Supplementary Figure 11

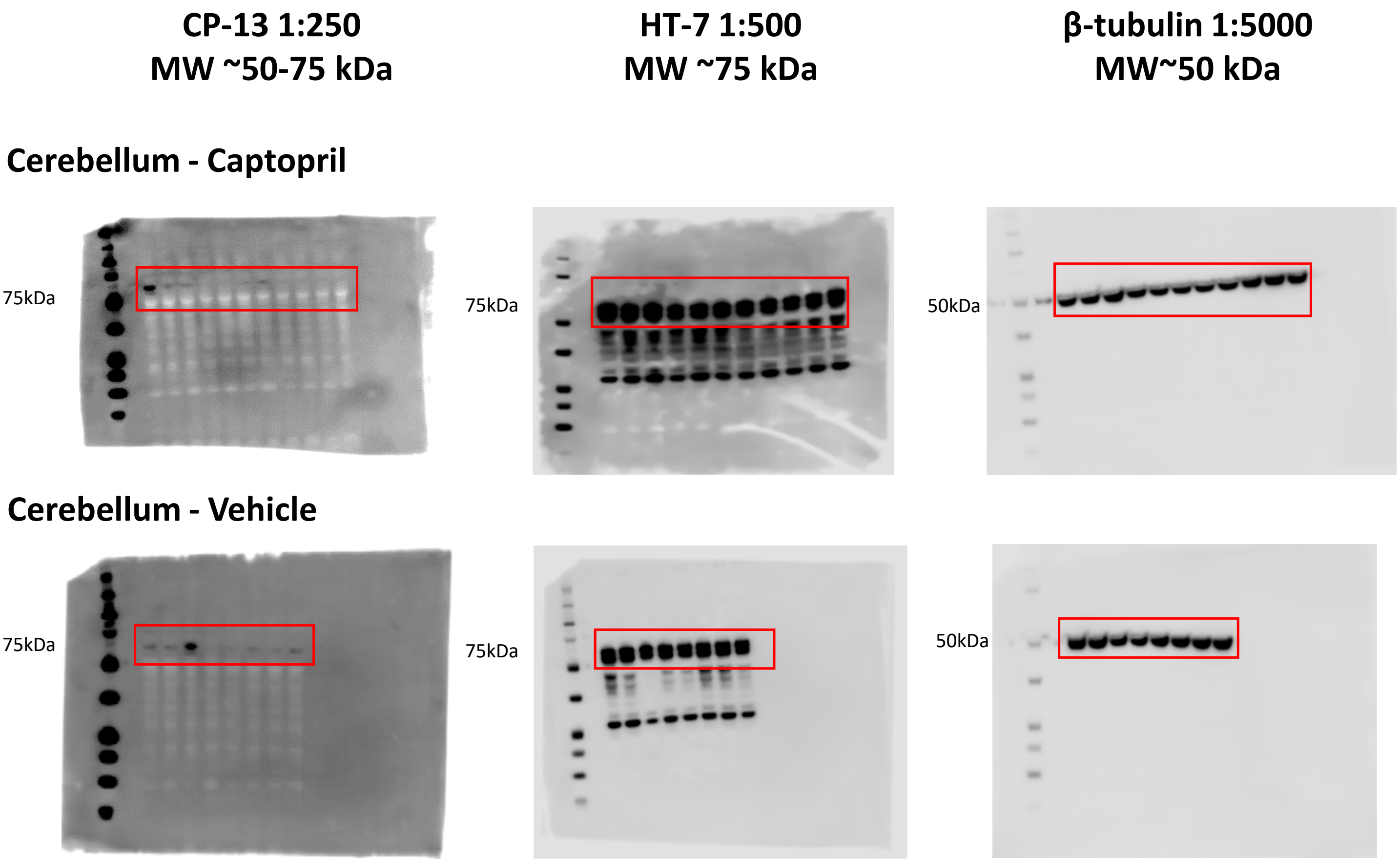

Supplementary Figure 12

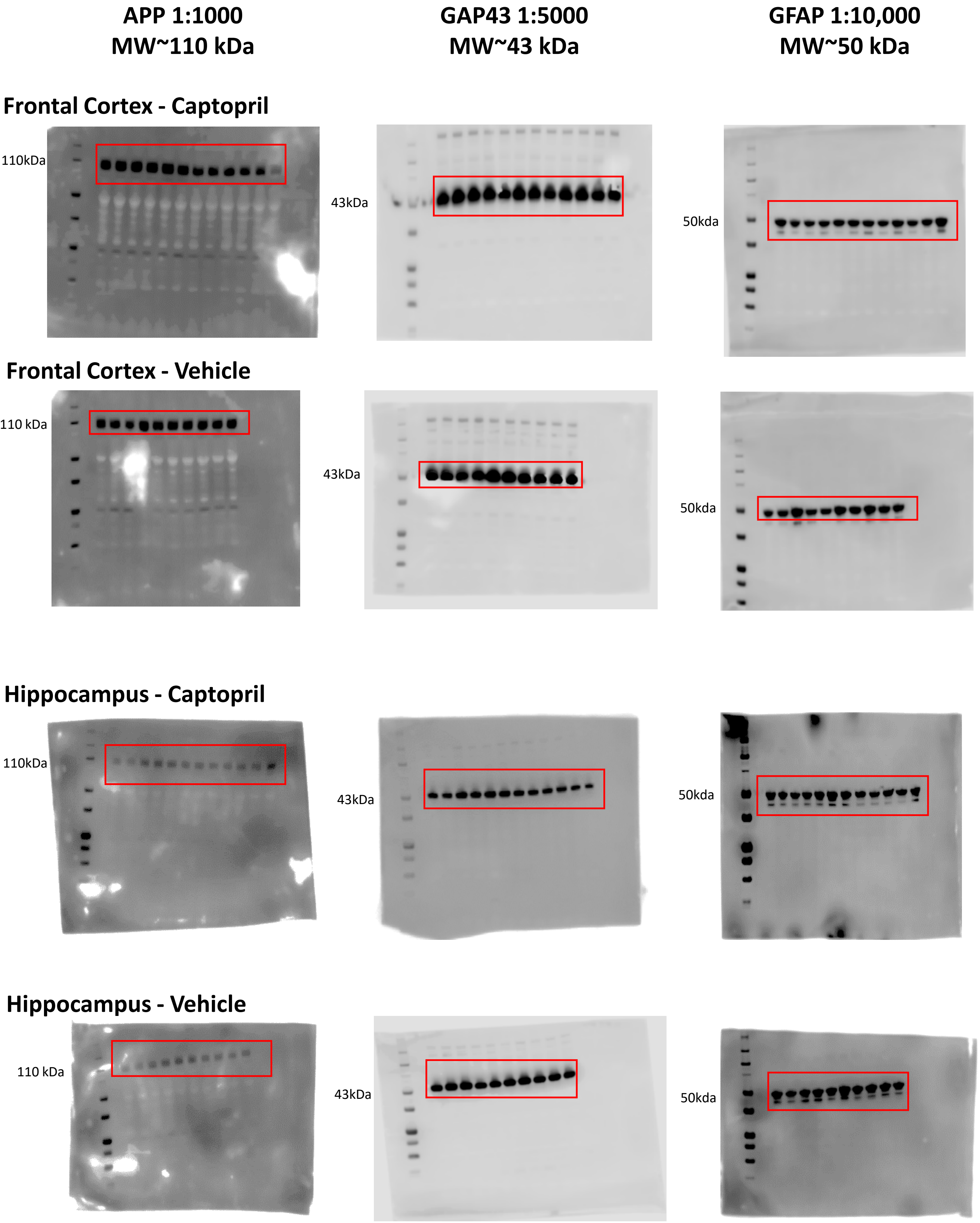

Supplementary Figure 13

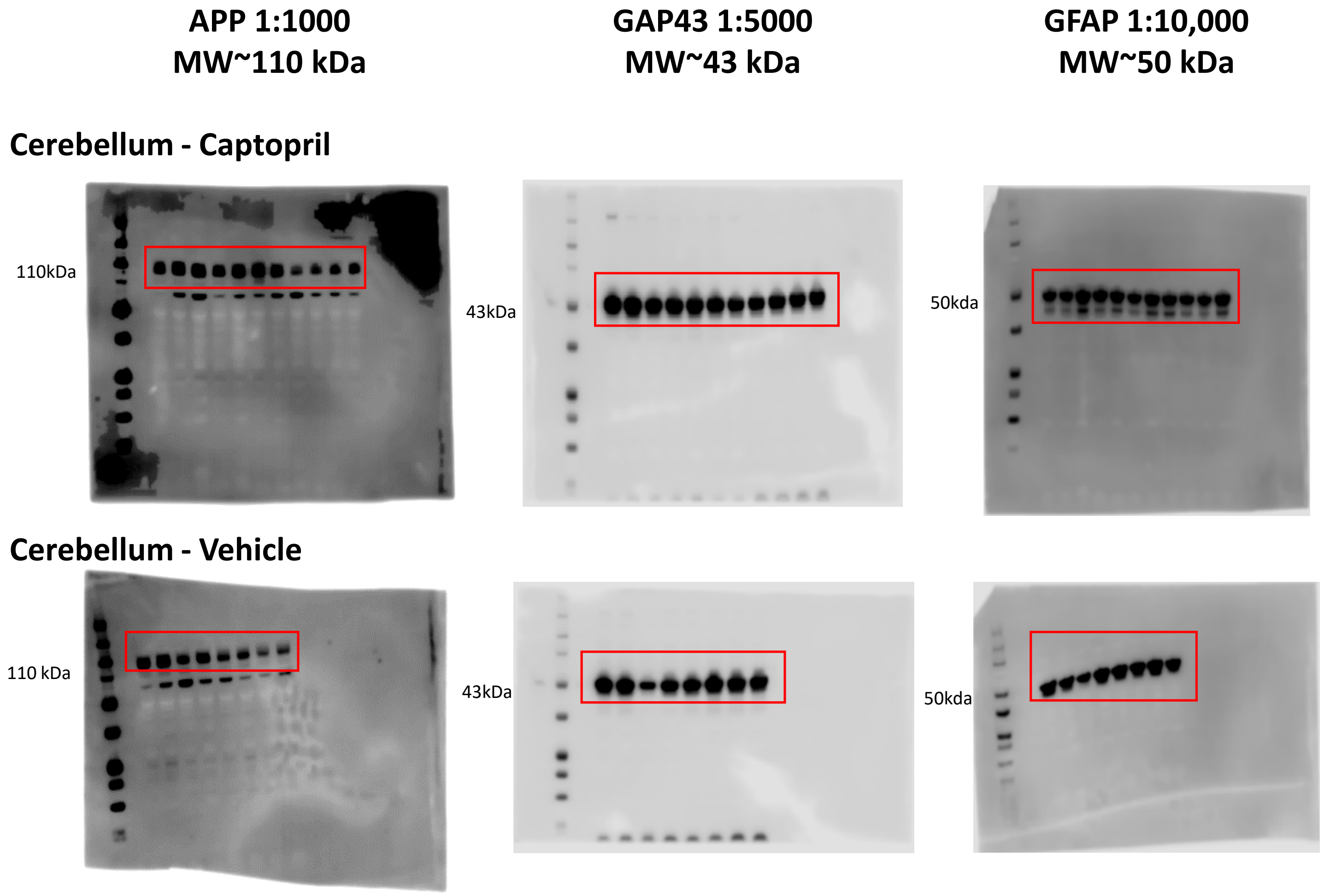

Supplementary Figure 14

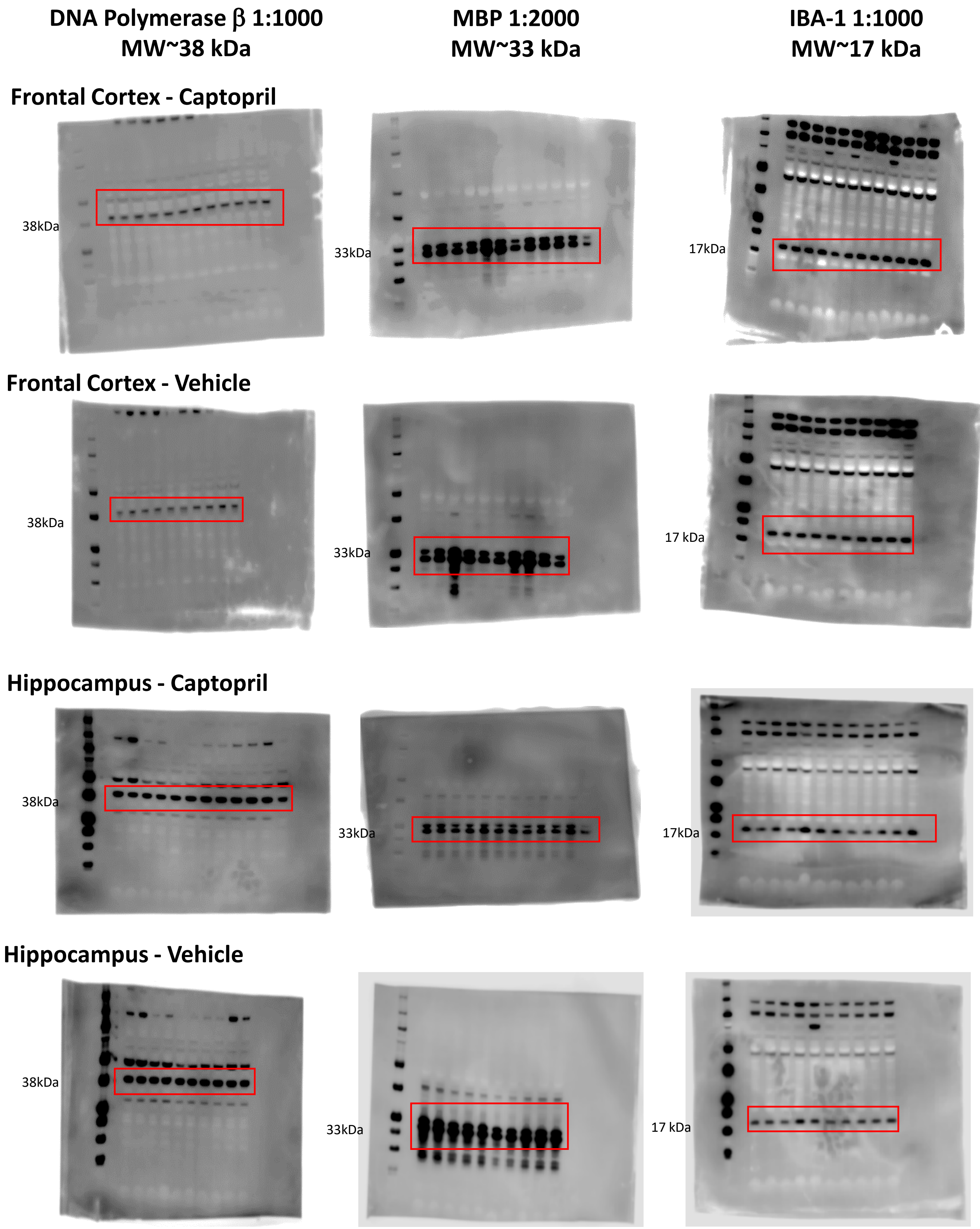

Supplementary Figure 15

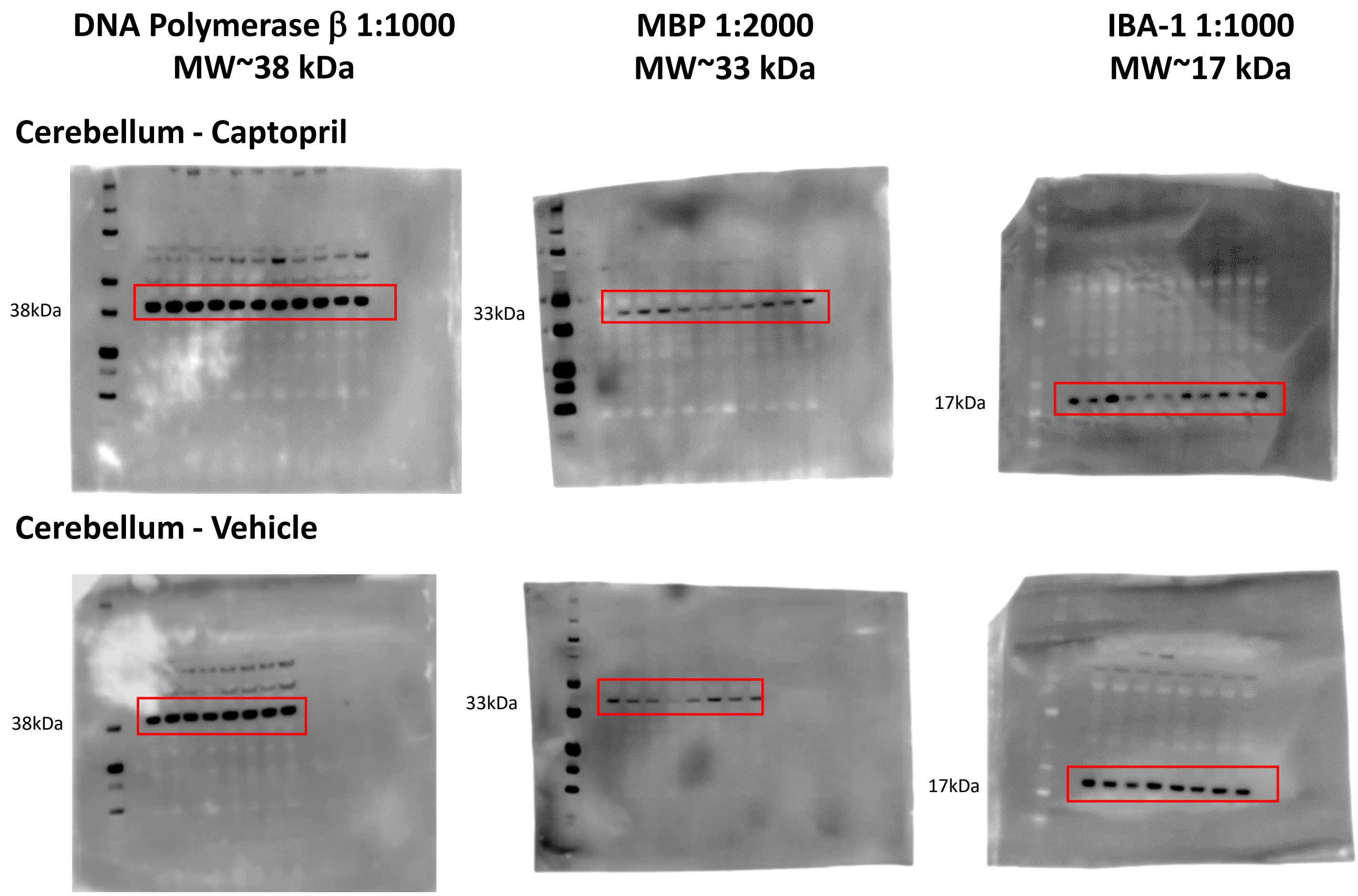

Supplementary Figure 16

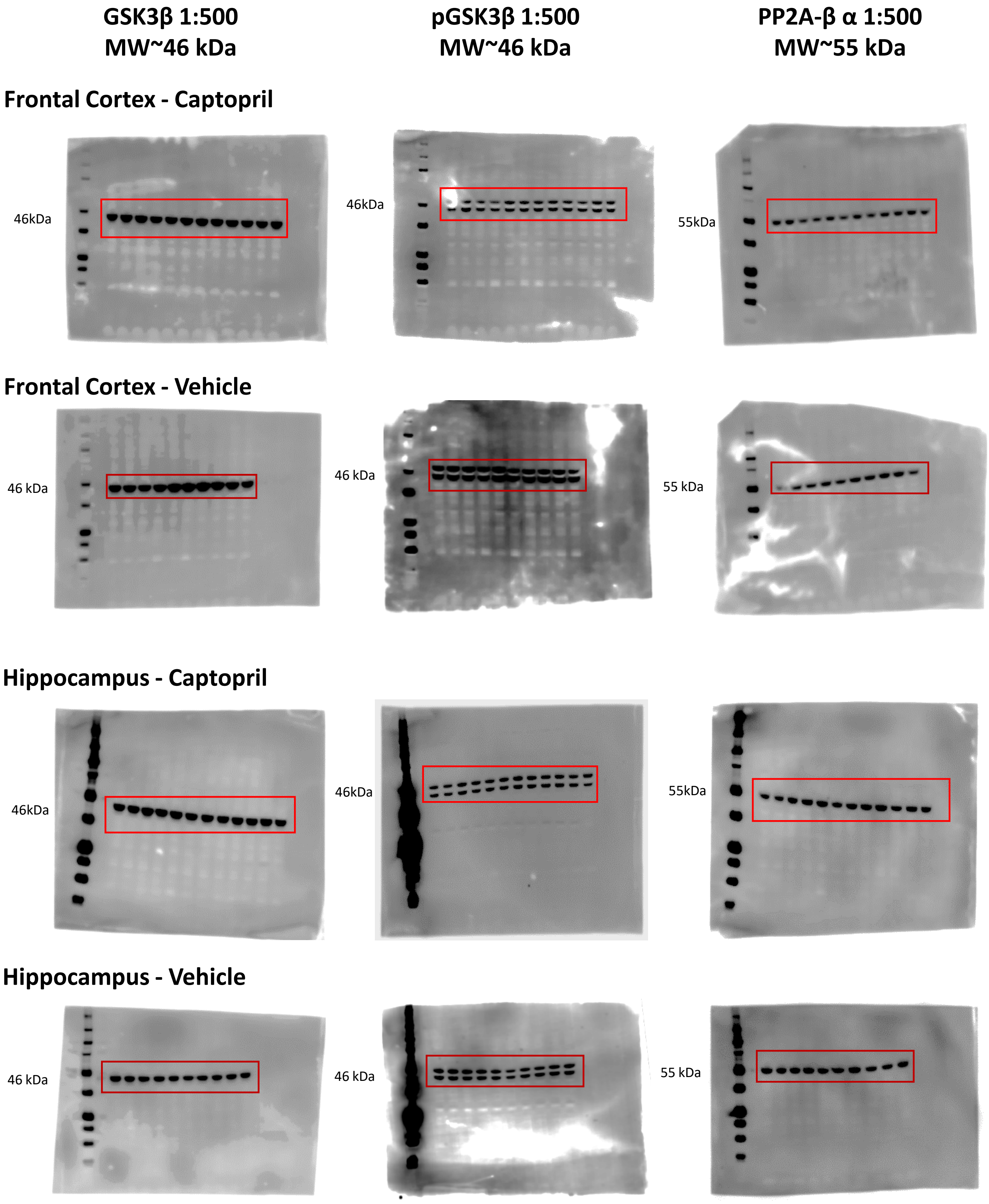

Supplementary Figure 17

GSK3β 1:500
MW~46 kDa

pGSK3β 1:500
MW~46 kDa

PP2A-β α 1:500
MW~55 kDa

Cerebellum - Captopril

46kDa

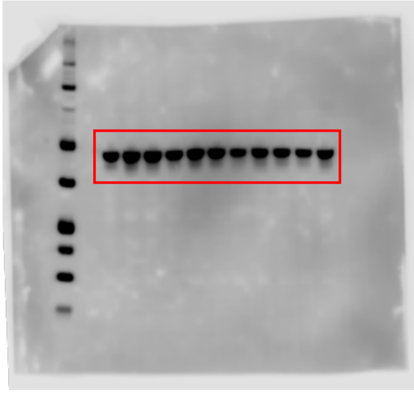

46kDa

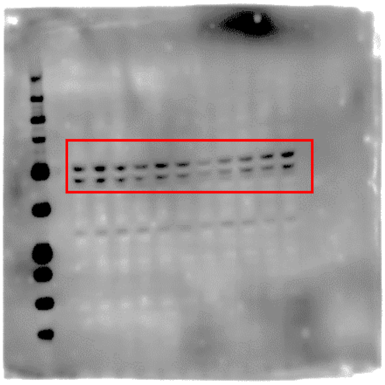

55kDa

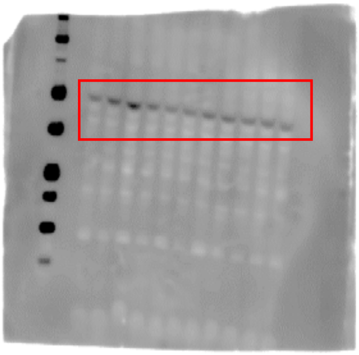

Cerebellum - Vehicle

46 kDa

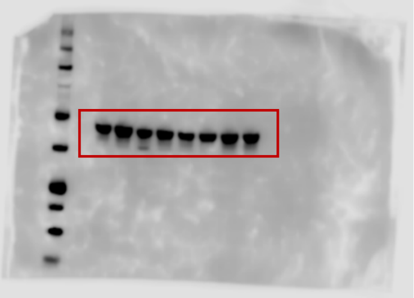

46 kDa

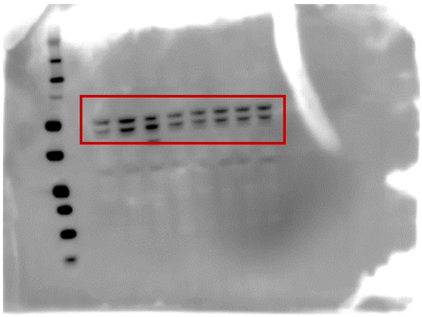

55 kDa

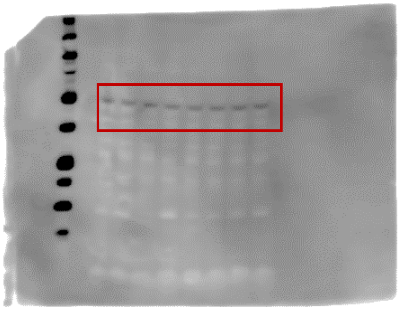

Supplementary Figure 18

GAPDH 1:40,000
MW-37 kDa
For CP13 primary

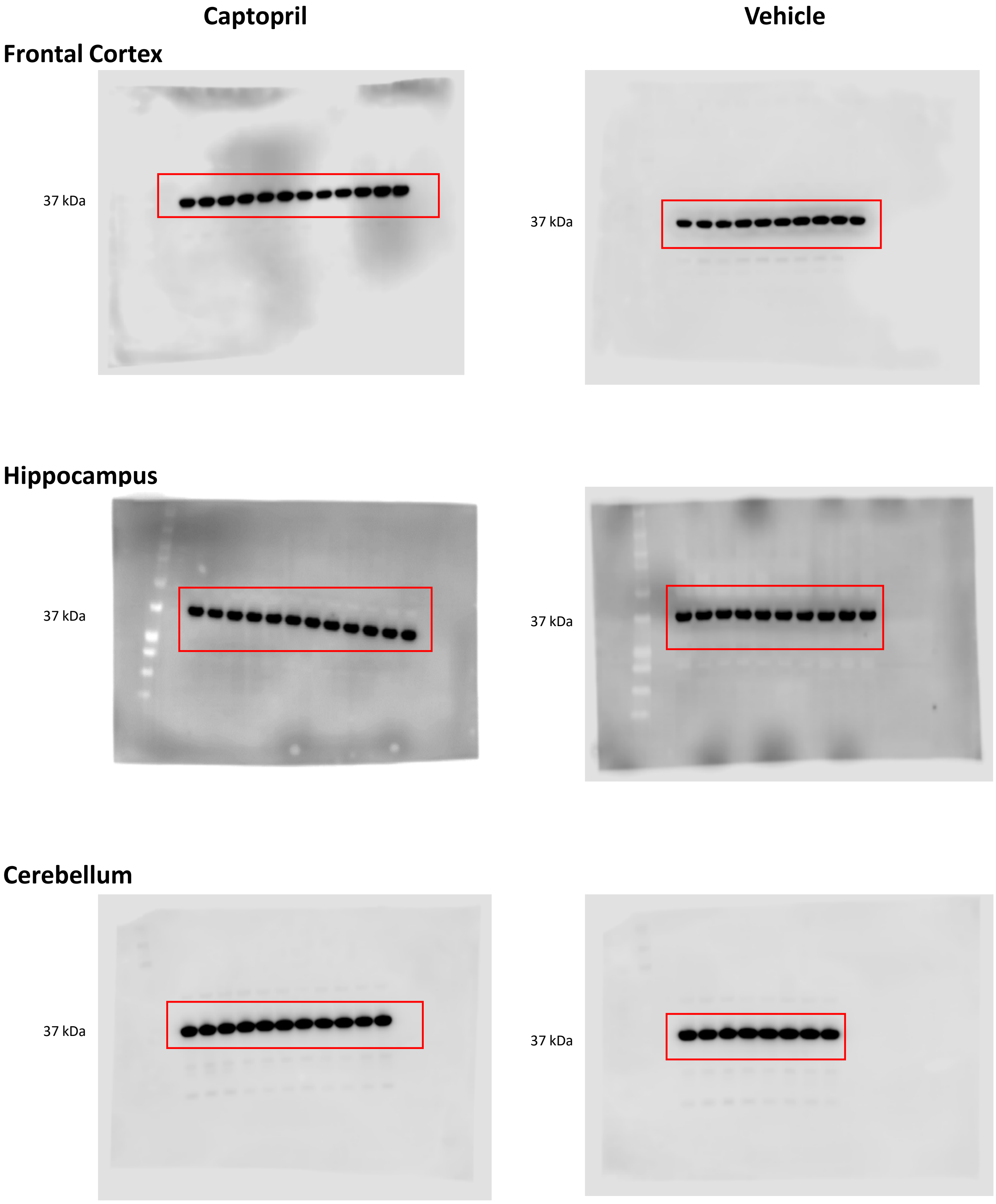

Supplementary Figure 19

GAPDH 1:40,000
MW-37 kDa
For HT7 primary

Captopril | **Vehicle**

Frontal Cortex

37 kDa

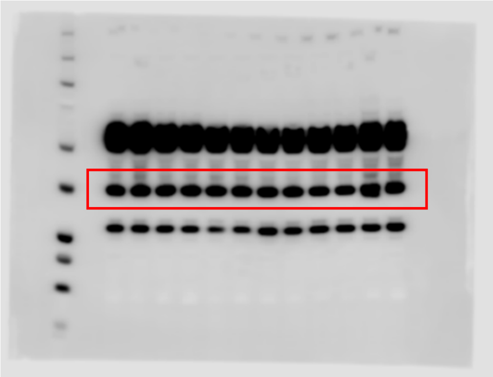

37 kDa

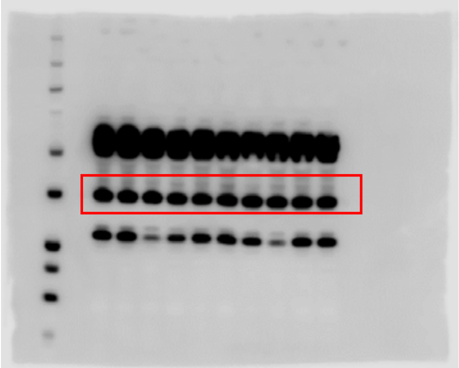

Hippocampus

37 kDa

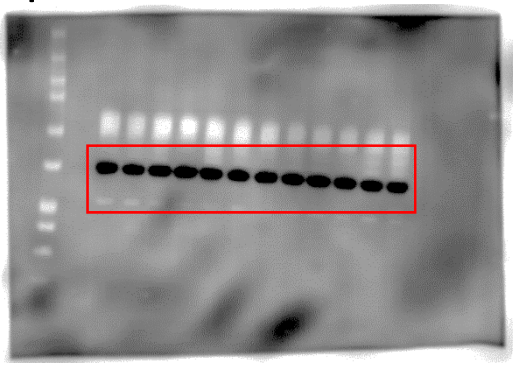

37 kDa

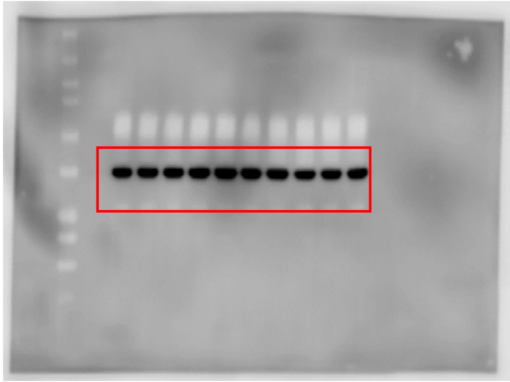

Cerebellum

37 kDa

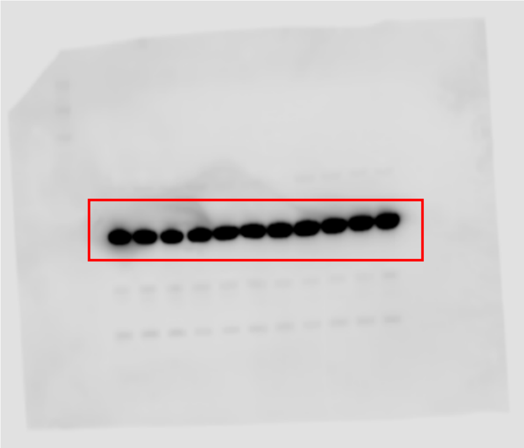

37 kDa

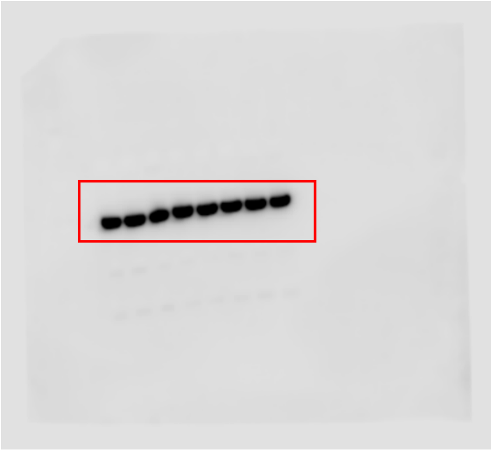

Supplementary Figure 20

GAPDH 1:40,000
MW-37 kDa
For βTubulin primary

Captopril | **Vehicle**

Frontal Cortex

37 kDa
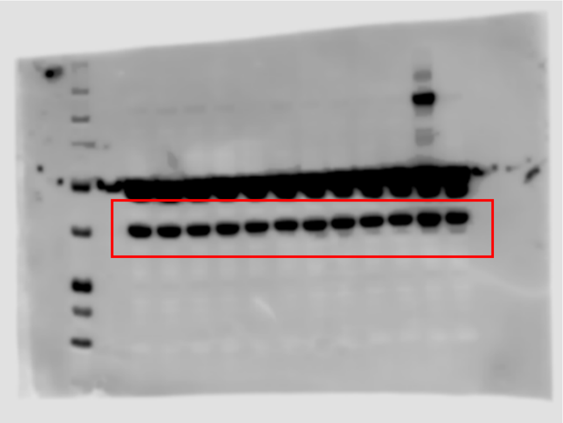

37 kDa
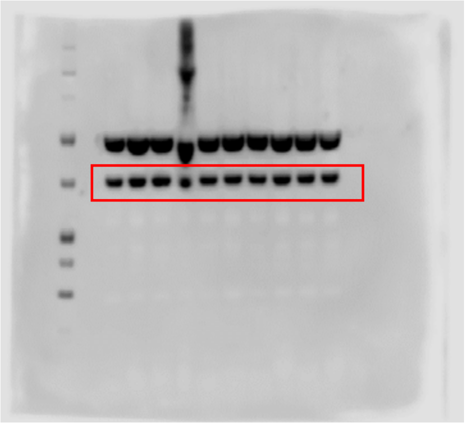

Hippocampus

37 kDa
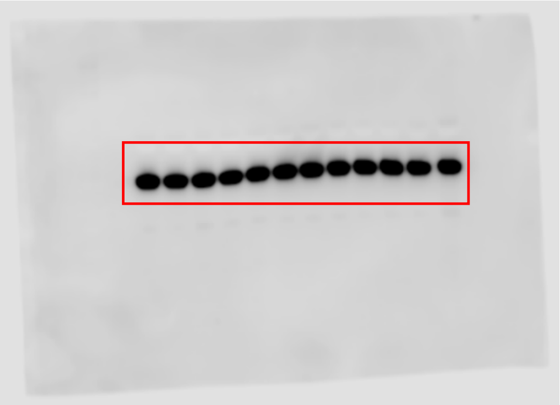

37 kDa
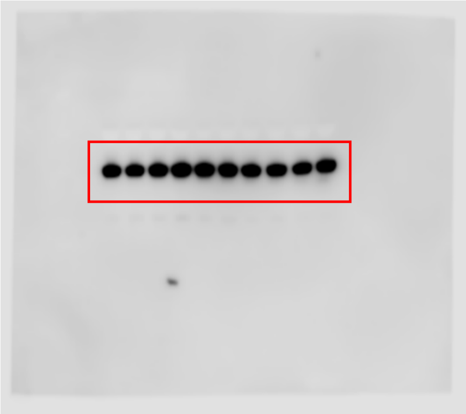

Cerebellum

37 kDa
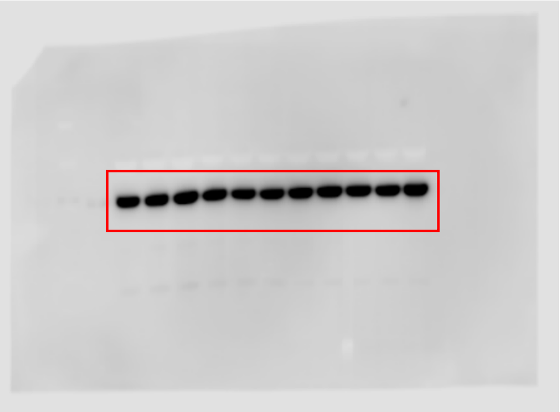

37 kDa
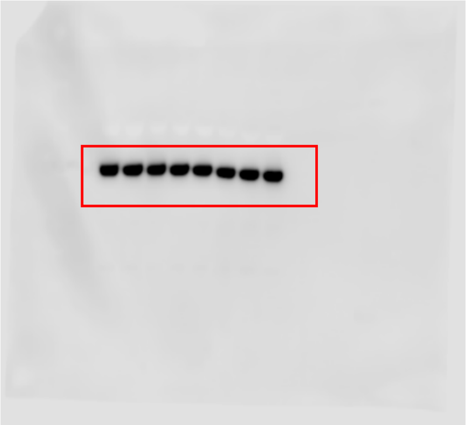

Supplementary Figure 21

GAPDH 1:40,000
MW-37 kDa
For APP primary

Captopril | **Vehicle**

Frontal Cortex

37 kDa
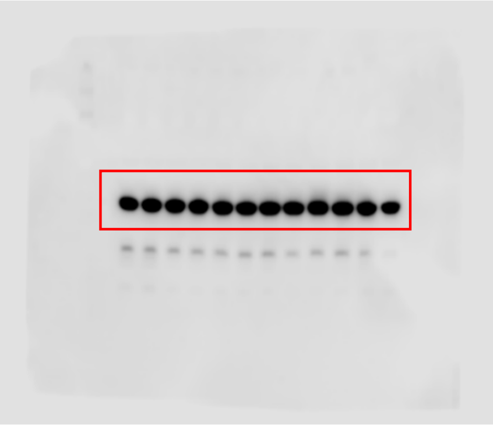

37 kDa
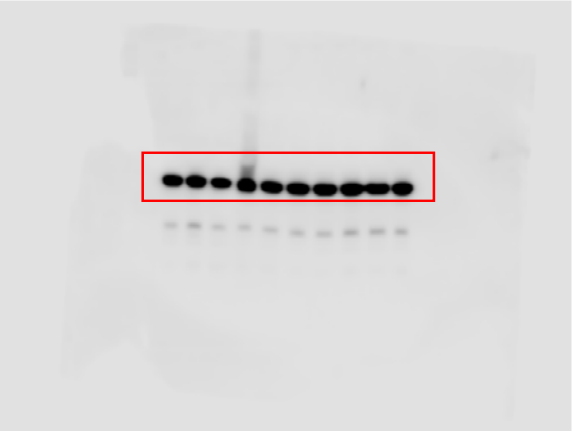

Hippocampus

37 kDa
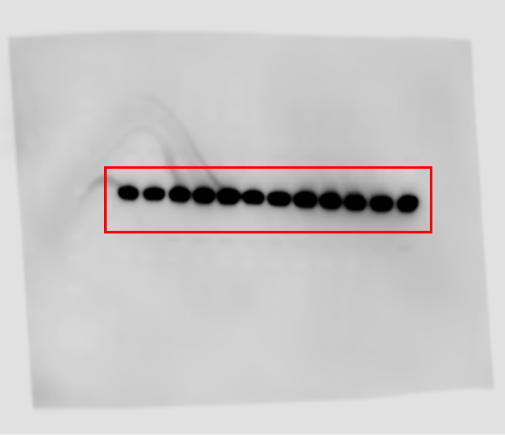

37 kDa
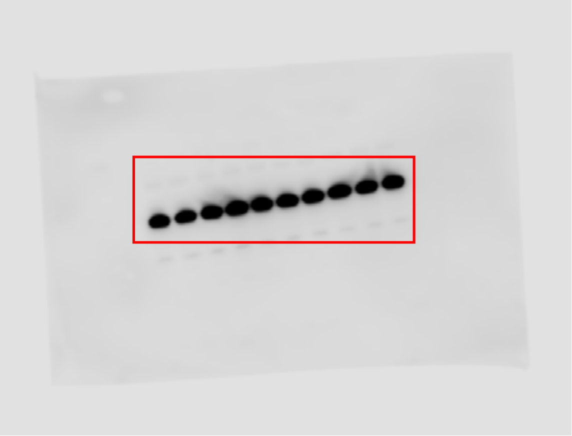

Cerebellum

37 kDa
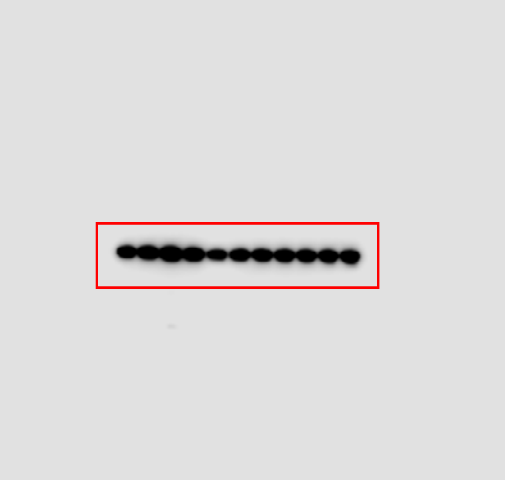

37 kDa
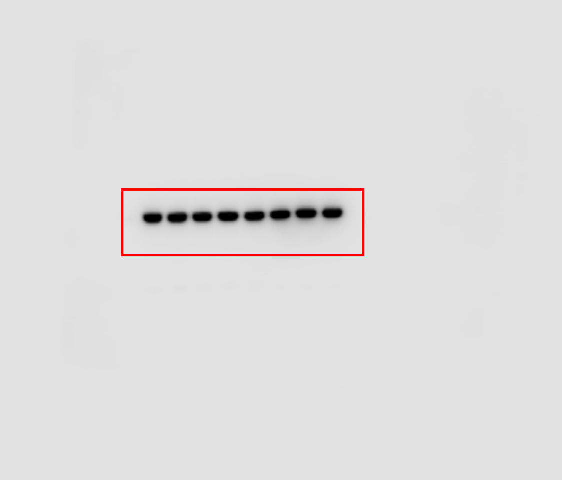

Supplementary Figure 22

GAPDH 1:40,000
MW-37 kDa
For GAP43 primary

Captopril **Vehicle**

Frontal Cortex

37 kDa

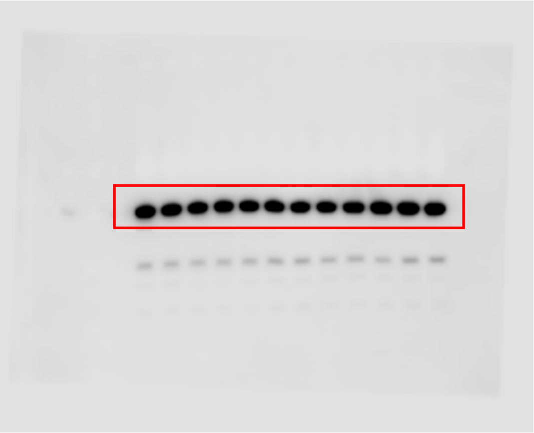

37 kDa

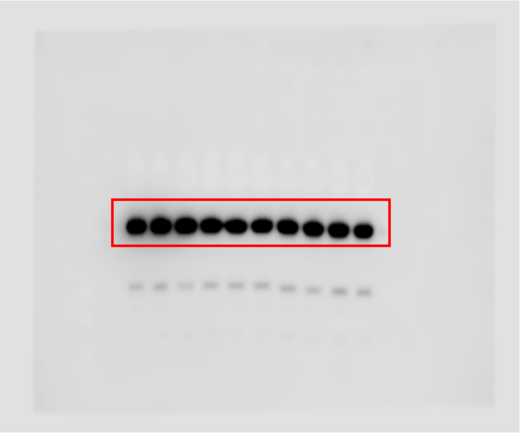

Hippocampus

37 kDa

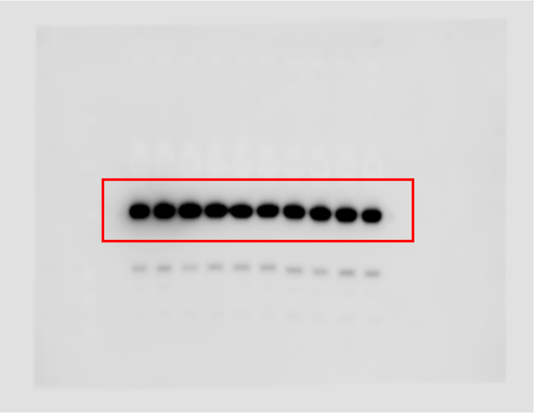

37 kDa

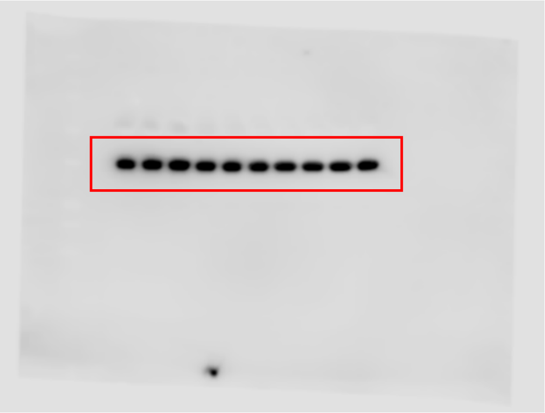

Cerebellum

37 kDa

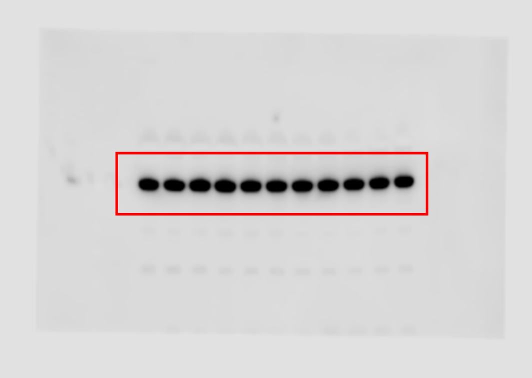

37 kDa

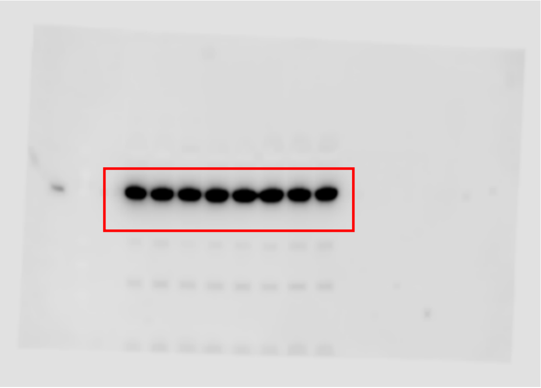

Supplementary Figure 23

GAPDH 1:40,000
MW-37 kDa
For GFAP primary

Captopril | **Vehicle**

Frontal Cortex

37 kDa

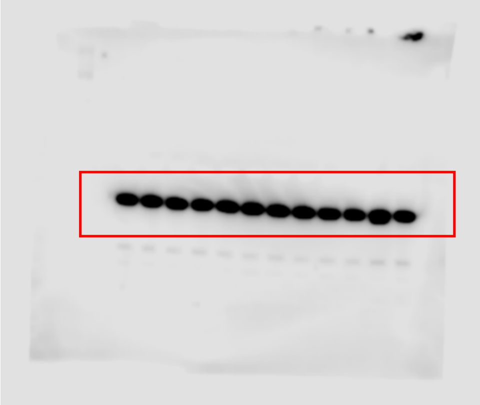

37 kDa

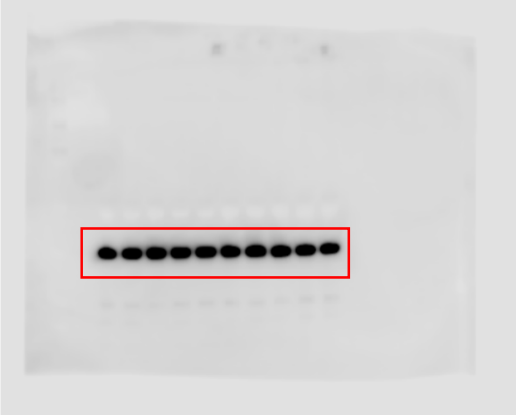

Hippocampus

37 kDa

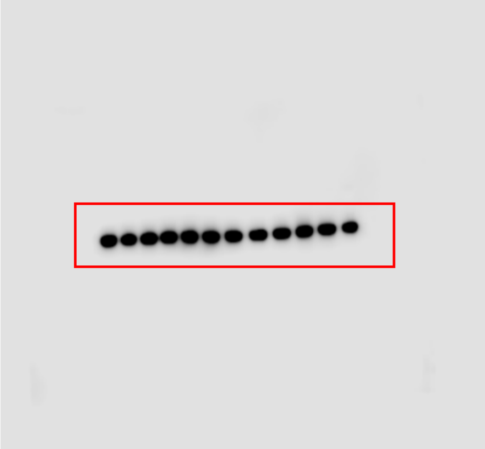

37 kDa

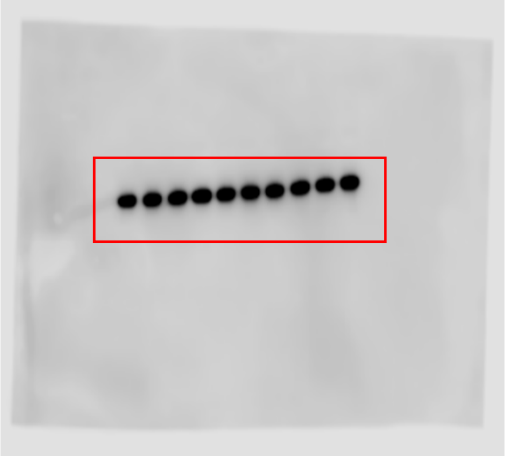

Cerebellum

37 kDa

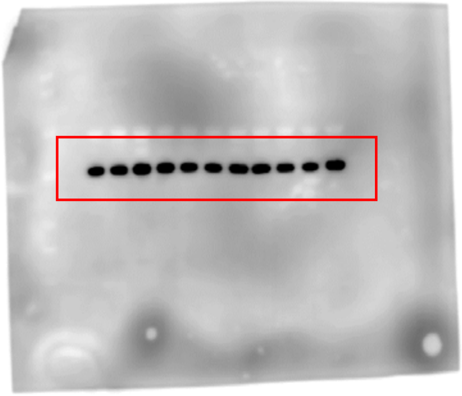

37 kDa

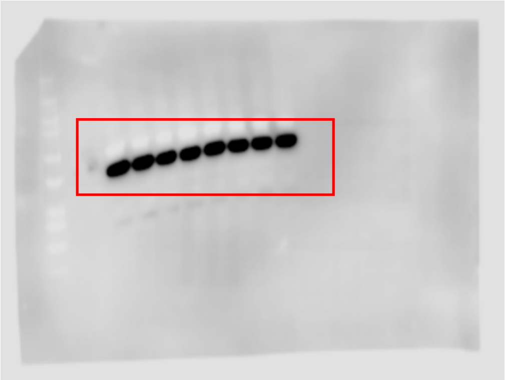

Supplementary Figure 24

GAPDH 1:40,000
MW-37 kDa
For DNA Polymerase β primary

Captopril **Vehicle**

Frontal Cortex

37 kDa

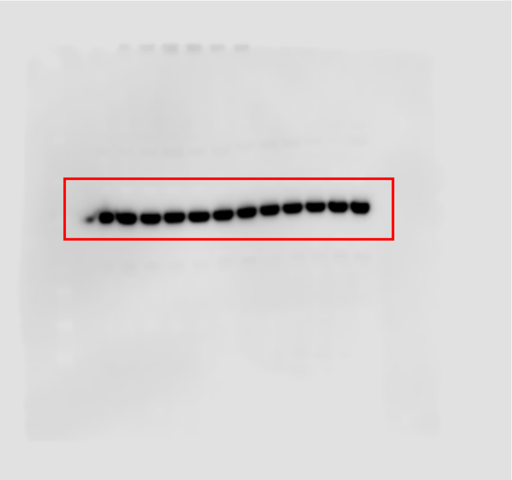

37 kDa

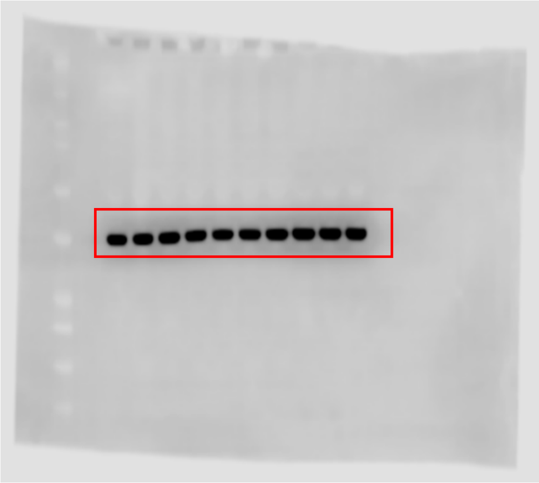

Hippocampus

37 kDa

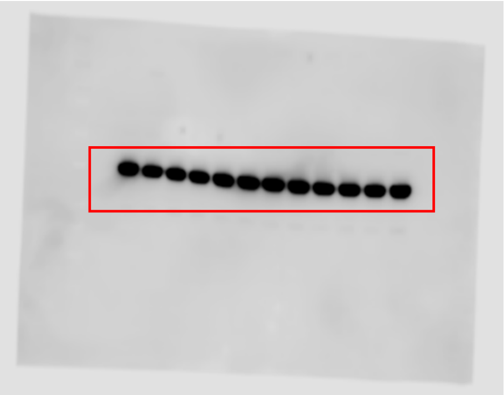

37 kDa

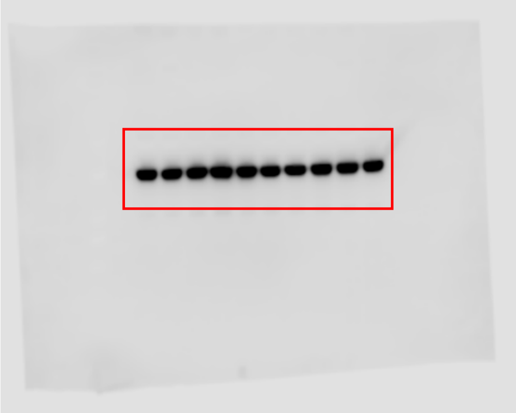

Cerebellum

37 kDa

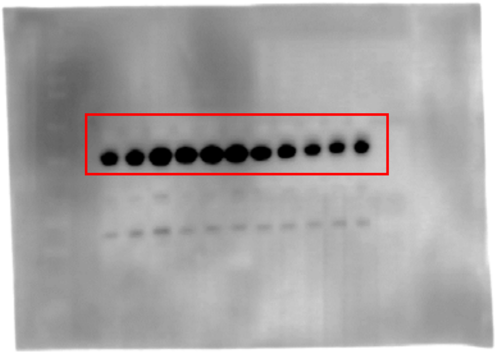

37 kDa

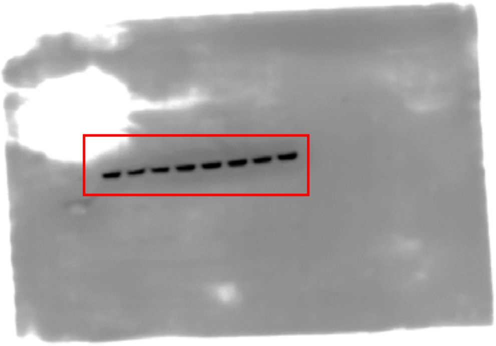

Supplementary Figure 25

GAPDH 1:40,000
MW-37 kDa
For MBP primary

Captopril | **Vehicle**

Frontal Cortex

37 kDa

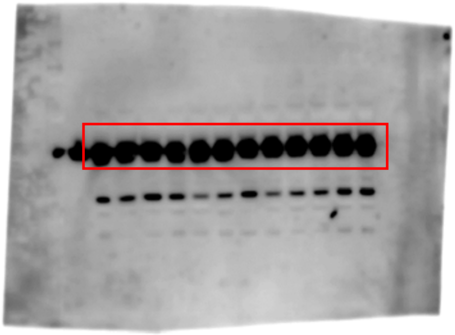

37 kDa

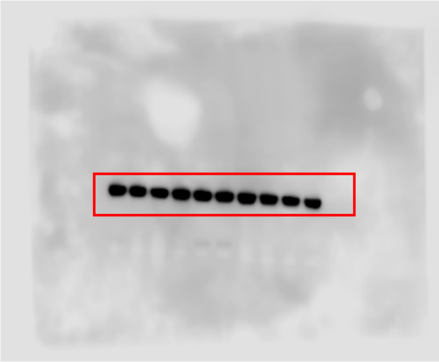

Hippocampus

37 kDa

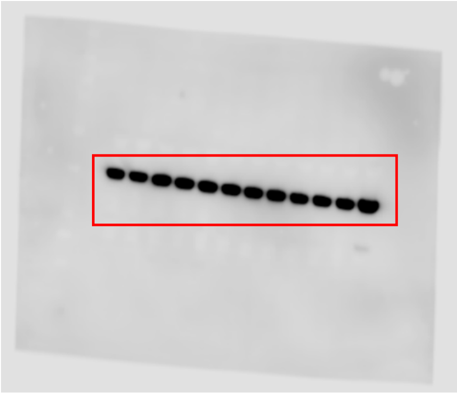

37 kDa

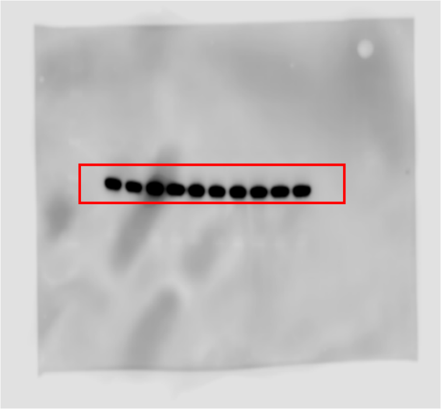

Cerebellum

37 kDa

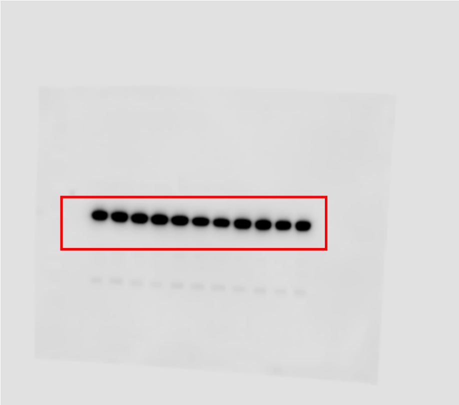

37 kDa

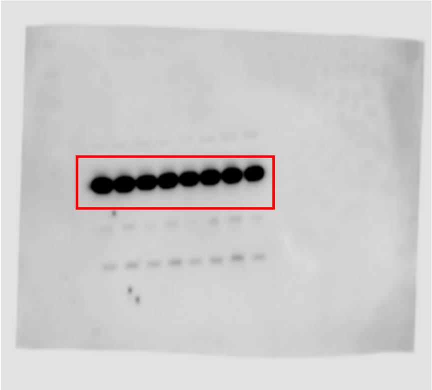

Supplementary Figure 26

GAPDH 1:40,000
MW-37 kDa
For IBA-1 primary

Captopril **Vehicle**

Frontal Cortex

37 kDa

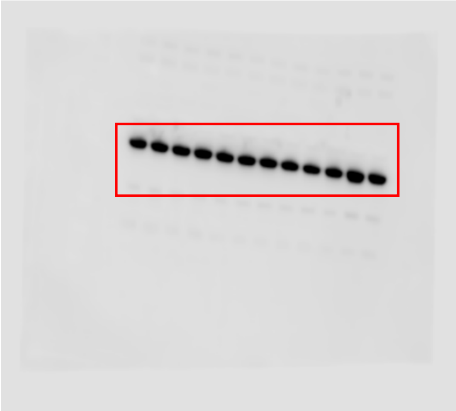

37 kDa

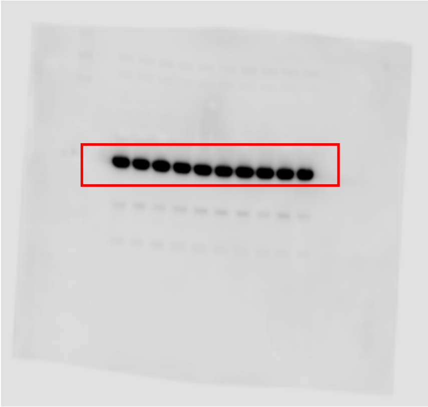

Hippocampus

37 kDa

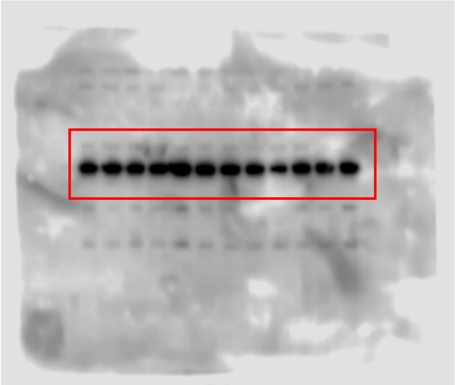

37 kDa

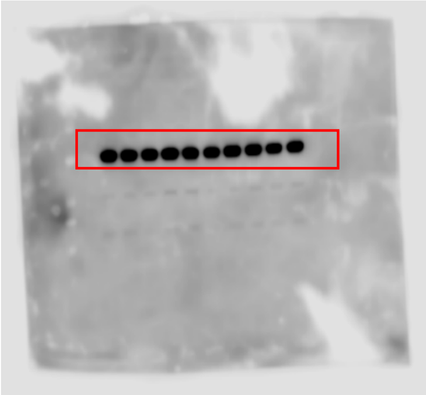

Cerebellum

37 kDa

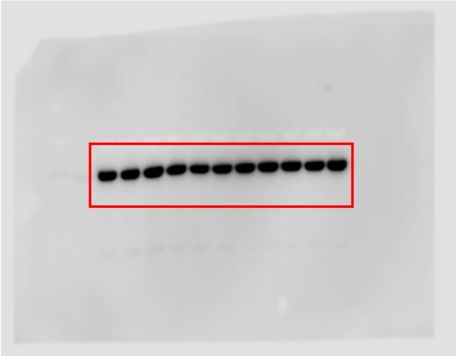

37 kDa

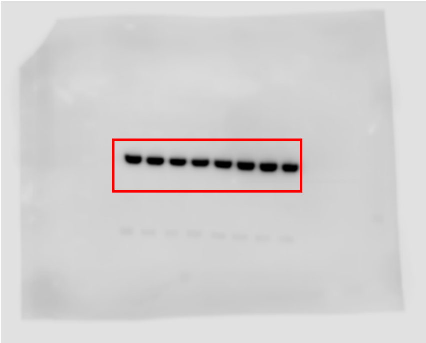

Supplementary Figure 27

GAPDH 1:40,000
MW-37 kDa
For GSK3β primary

Captopril **Vehicle**

Frontal Cortex

37 kDa
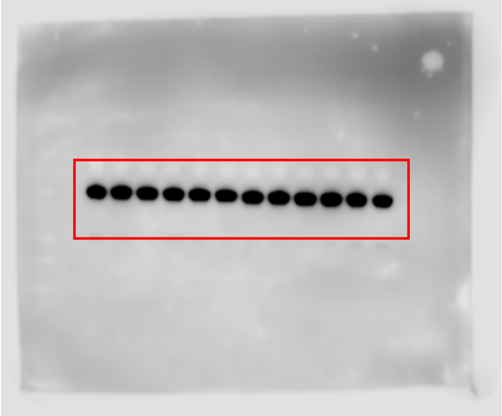

37 kDa
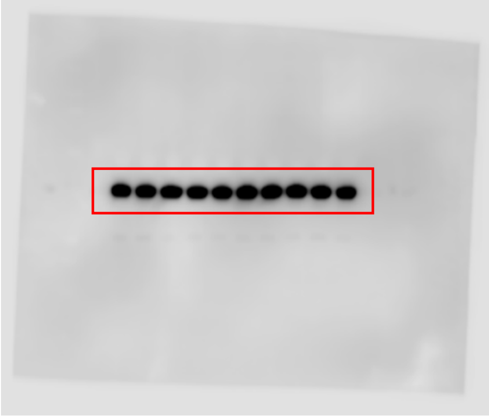

Hippocampus

37 kDa
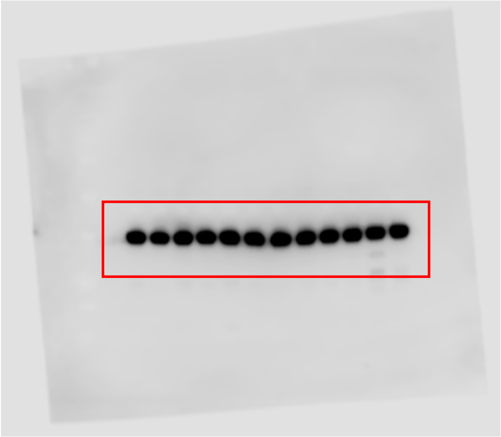

37 kDa
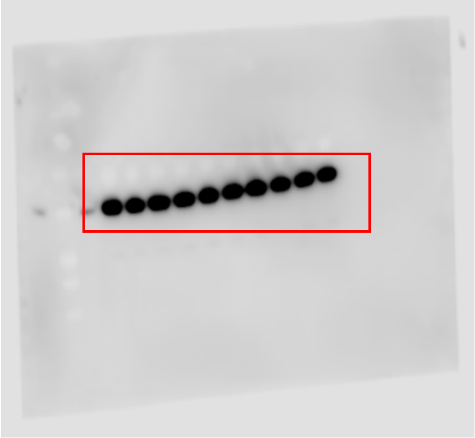

Cerebellum

37 kDa
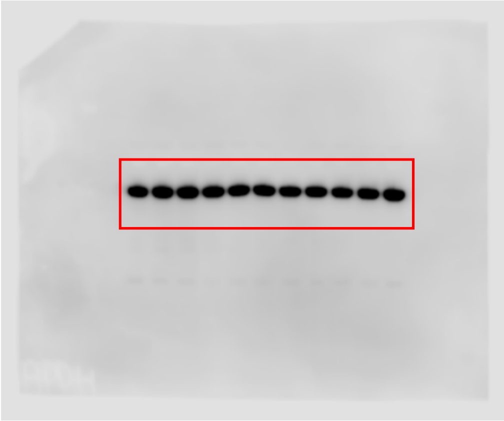

37 kDa
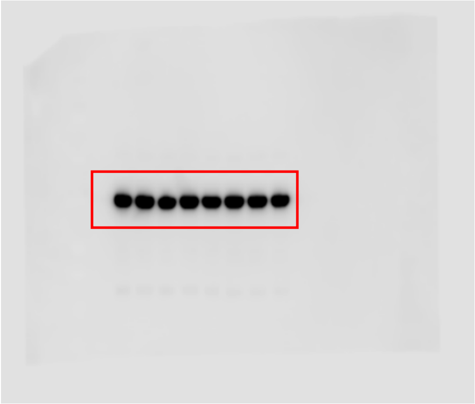

Supplementary Figure 28

GAPDH 1:40,000
MW-37 kDa
For pGSK3β primary

Captopril | **Vehicle**

Frontal Cortex

37 kDa

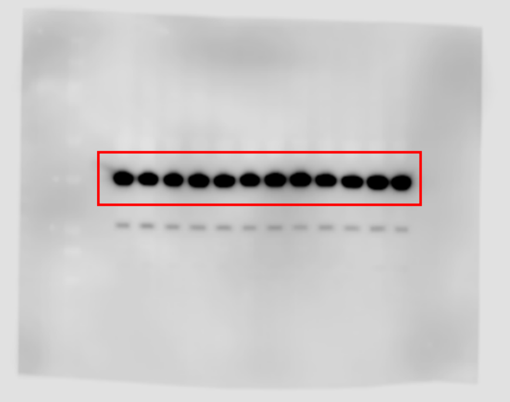

37 kDa

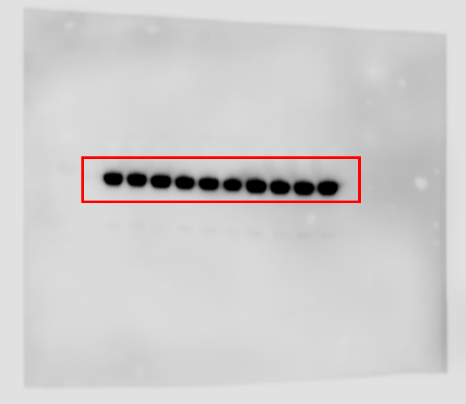

Hippocampus

37 kDa

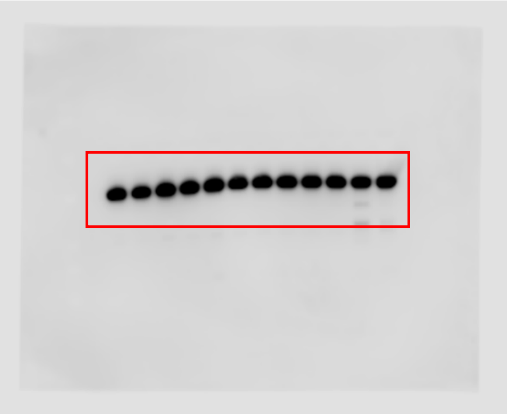

37 kDa

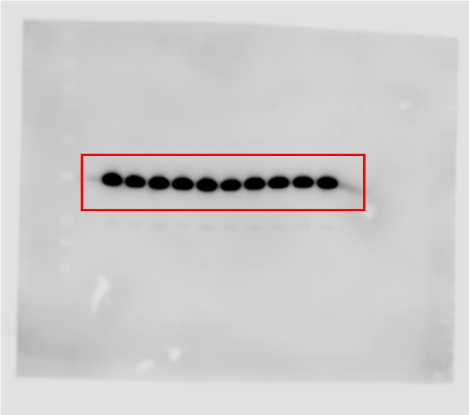

Cerebellum

37 kDa

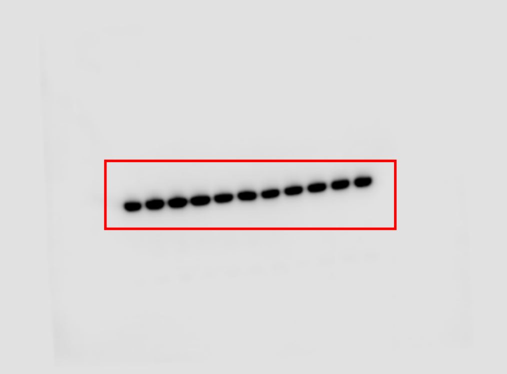

37 kDa

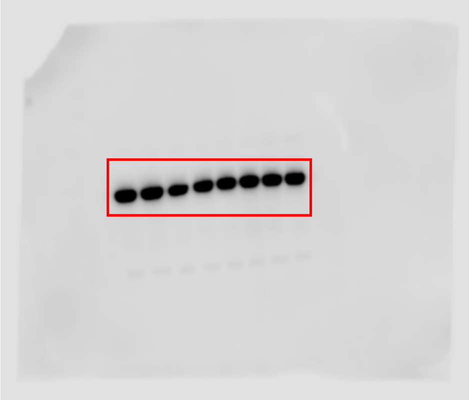

Supplementary Figure 29

GAPDH 1:40,000
MW-37 kDa
For PP2Aβα primary

Captopril | **Vehicle**

Frontal Cortex

37 kDa

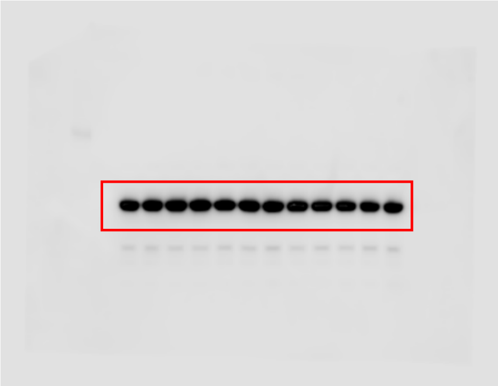

37 kDa

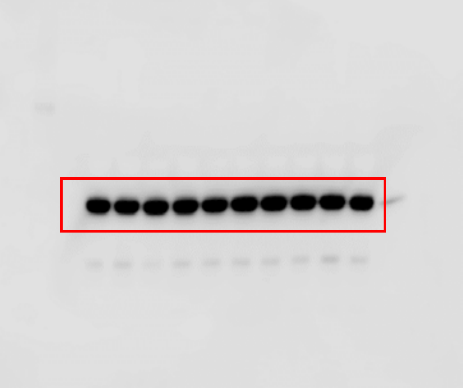

Hippocampus

37 kDa

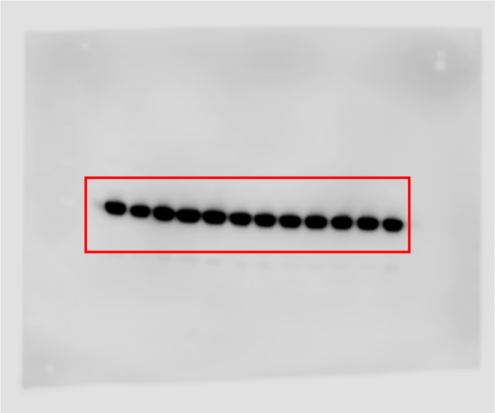

37 kDa

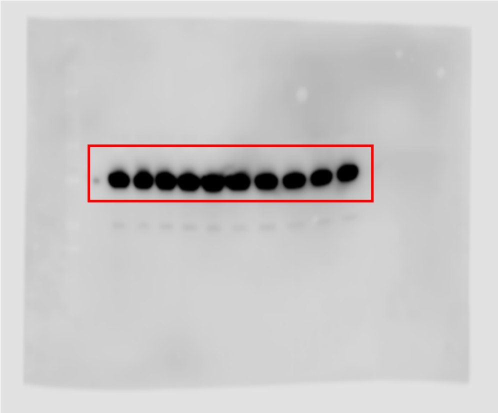

Cerebellum

37 kDa

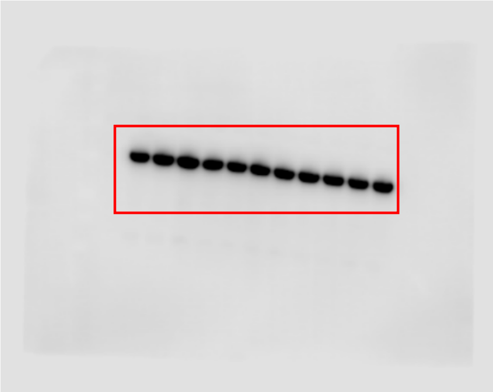

37 kDa

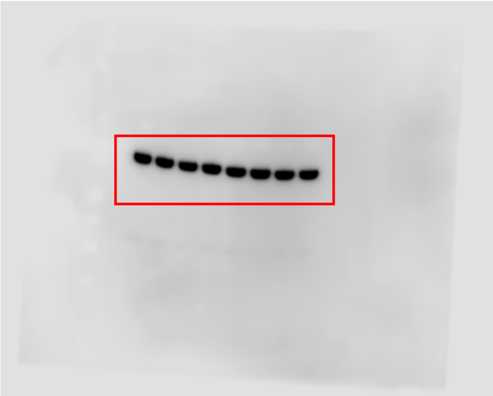

Supplementary Figure 30

ClinicalTrials.gov Search Results 09/30/2020

	Title	Status	Study Results	Conditions	Interventions	Locations
1	Study of Low Dose Whole Brain Irradiation in the Treatment of Alzheimer's Disease	Recruiting	No Results Available	• Alzheimer's Disease	• Radiation: 5 daily fractions of 2 Gy<br>• Radiation: 10 daily fractions of 2 Gy	• Beaumont Health, Farmington Hills, Michigan, United States<br>• Beaumont Health, Royal Oak, Michigan, United States
2	The Safety and Scientific Validity of Low-dose Whole Brain Radiotherapy in Alzheimer's Disease.	Recruiting	No Results Available	• Alzheimer Disease	• Radiation: low dose whole brain radiation to treat Alzheimer disease	• Kyung Hee University Hospital at Gangdong, Seoul, Korea, Republic of
3	Low Dose Ionizing Radiation Using CT Scans as a Potential Therapy for Alzheimer's Dementia: A Pilot Study	Recruiting	No Results Available	• Dementia Alzheimers	• Radiation: CT scan	• Baycrest Health Sciences, Toronto, Ontario, Canada
4	Effect of Low Dose Radiotherapy on Brain Amyloidosis in the Treatment of Alzheimer's Disease	Recruiting	No Results Available	• Alzheimer Disease	• Radiation: Low dose radiotherapy	• Geneva University Hospital, Geneva 14, Switzerland
5	Evaluation of ZK 6032924 in Probable Alzheimer's Disease Patients Versus Healthy Volunteers and the Radiation Dosimetry of ZK 6032924 in Healthy Volunteers	Completed	No Results Available	• Positron-Emission Tomography<br>• Alzheimer's Disease	• Drug: F-18 FEDAA1106 (BAY85-8101)	• Amsterdam, Netherlands<br>• Stockholm, Sweden<br>• Stockholm, Sweden
6	Safety, Radiation Dosimetry, Biokinetics, and Effectiveness of [18F]MK3328 (MK-3328-001)	Completed	Has Results	• Alzheimer's Disease	• Drug: [18F]MK-3328	
7	Evaluation of the Diagnostic Potential of BAY1006578 in Probable Alzheimers Disease Patients Versus Healthy Volunteers and Radiation Dosimetry of BAY1006578 in Healthy Volunteers	Completed	No Results Available	• Diagnostic Imaging	• Drug: BAY1006578	• Turku, Finland<br>• Stockholm, Sweden
8	Evaluation of the Neuroinflammation Pattern of BAY85-8102 F-18, DPA-714 in Probable Alzheimers Disease Patients Versus Healthy Volunteers and Radiation Dosimetry of F 18, DPA-714 in Healthy Volunteers	Completed	No Results Available	• Diagnostic Imaging	• Drug: F-18 DPA-714 (BAY85-8102)	• Turku, Finland<br>• Amsterdam, Netherlands
9	Far Infrared Treatment for Alzheimer's Disease	Unknown status	No Results Available	• Alzheimer Disease	• Radiation: Far Infrared Radiation (5µm to 20µm wavelength)	• The Centre for Incurable Diseases, Toronto, Ontario, Canada
10	Low Dose RT to Reduce Cerebral Amyloidosis in Early Alzheimer's	Active, not recruiting	No Results Available	• Alzheimer's Disease	• Radiation: 10 GY in 5 daily fractions<br>• Radiation: 20 GY in 10 daily fractions	• Virginia Commonwealth University, Richmond, Virginia, United States
11	Initial Investigation of [18F]P16-129 in Alzheimer's Disease Patients and Healthy Volunteers	Active, not recruiting	No Results Available	• Alzheimer Disease	• Drug: [18F]P16-129	• Johns Hopkins Medical Institutions, Baltimore, Maryland, United States
12	Augmenting Flortaucipir Dosimetry Estimates	Completed	Has Results	• Alzheimer's Disease	• Drug: Flortaucipir F18	• Molecular NeuroImaging, New Haven, Connecticut, United States
13	Low Level Laser Therapy Impact on Cognitive Function and Quality of Life in Alzheimer Anemic Elderly Patients	Completed	No Results Available	• Alzheimer Disease<br>• Cognitive Impairment, Mild	• Device: low level laser therapy (laser acupuncture combined with nasal laser irradiation)<br>• Other: aerobic exercise	• Cairo, Giza, Dokki, Egypt

U.S. National Library of Medicine | U.S. National Institutes of Health | U.S. Department of Health & Human Services

Supplementary Figure 31

Supplementary Figure 1

Weight over time.

No significant differences in weight gain were noted between SH (n=10) and RAD (n=12) over the course of the study.

Supplementary Figure 2

Comparison of Vehicle and Captopril Administration in SH and RAD groups for CP13 and HT7

Scatter plot representing the densitometric ratio of levels of CP13 (**A**.) and HT7 (**B**.) with respect to GAPDH as measured in the frontal cortex, hippocampus and cerebellum in the brains of Vehicle or treated, Gottingen mini-pigs 30-days after total body radiation (1.79 Gy of Cobalt [^{60}Co]) or Sham radiation. One-way ANOVA confirmed there was no statistical difference between protein expressions in captopril or vehicle treated groups thus, Sham + Captopril and Sham + Vehicle samples were pooled and Radiation + Captopril and Radiation + Vehicle were pooled for overall data analysis.

Supplementary Figure 3

Comparison of Vehicle and Captopril Administration in SH and RAD groups for βTubulin and APP

Scatter plot representing the densitometric ratio of levels of βTubulin (**A**.) and APP (**B**.) with respect to GAPDH as measured in the frontal cortex, hippocampus and cerebellum in the brains of vehicle or captopril treated, Gottingen mini-pigs 30-days after total body radiation (1.79 Gy of Cobalt [^{60}Co]) or Sham radiation. One-way ANOVA confirmed there was no statistical difference between protein expressions in captopril or vehicle treated groups thus, Sham +

Captopril and Sham + Vehicle samples were pooled and Radiation + Captopril and Radiation + Vehicle were pooled for overall data analysis.

Supplementary Figure 4

Comparison of Vehicle and Captopril Administration in SH and RAD groups for GAP43 and GFAP

Scatter plot representing the densitometric ratio of levels of GAP43 (**A**.) and GFAP (**B**.) with respect to GAPDH as measured in the frontal cortex, hippocampus and cerebellum in the brains of vehicle or captopril treated, Gottingen mini-pigs 30-days after total body radiation (1.79 Gy of Cobalt [^{60}Co]) or Sham radiation. One-way ANOVA confirmed there was no statistical difference between protein expressions in captopril or vehicle treated groups thus, Sham + Captopril and Sham + Vehicle samples were pooled and Radiation + Captopril and Radiation + Vehicle were pooled for overall data analysis.

Supplementary Figure 5

Comparison of Vehicle and Captopril Administration in SH and RAD groups for DNA polymerase-β and MBP

Scatter plot representing the densitometric ratio of levels of DNA Polymerase β (**A**.) and MBP (**B**.) with respect to GAPDH as measured in the frontal cortex, hippocampus and cerebellum in the brains of vehicle or captopril treated, Gottingen mini-pigs 30-days after total body radiation (1.79 Gy of Cobalt [^{60}Co]) or Sham radiation. One-way ANOVA confirmed there was no statistical difference between protein expressions in captopril or vehicle treated groups thus,

Sham + Captopril and Sham + Vehicle samples were pooled and Radiation + Captopril and Radiation + Vehicle were pooled for overall data analysis.

Supplementary Figure 6

Comparison of Vehicle and Captopril Administration in SH and RAD groups for IBA-1 and GSK3β

Scatter plot representing the densitometric ratio of levels of IBA-1(**A**.) and GSK3β (**B**.) with respect to GAPDH as measured in the frontal cortex, hippocampus and cerebellum in the brains of vehicle or captopril treated, Gottingen mini-pigs 30-days after total body radiation (1.79 Gy of Cobalt [^{60}Co]) or Sham radiation. One-way ANOVA confirmed there was no statistical difference between protein expressions in captopril or vehicle treated groups thus, Sham + Captopril and Sham + Vehicle samples were pooled and Radiation + Captopril and Radiation + Vehicle were pooled for overall data analysis.

Supplementary Figure 7

Comparison of Vehicle and Captopril Administration in SH and RAD groups for pGSK3β and PP2A-$\beta\alpha$

Scatter plot representing the densitometric ratio of levels of pGSK3β (**A**.) and PP2A-βα (**B**.) with respect to GAPDH as measured in the frontal cortex, hippocampus and cerebellum in the brains of vehicle or captopril treated, Gottingen mini-pigs 30-days after total body radiation (1.79 Gy of Cobalt [^{60}Co]) or Sham radiation. One-way ANOVA confirmed there was no statistical difference between protein expressions in captopril or vehicle treated groups thus,

Sham + Captopril and Sham + Vehicle samples were pooled and Radiation + Captopril and Radiation + Vehicle were pooled for overall data analysis.

Supplementary Figure 8

MBP and IBA-1 expression in brain following total body radiation.

Histograms representing the densitometric ratio of levels of MBP (**A**.) and IBA-1 (**B**.) with respect to GAPDH as measured in the frontal cortex, hippocampus and cerebellum in the brains of Gottingen mini-pigs 30-days after total body radiation (1.79 Gy of Cobalt (^{60}Co)) with representative western blots# for sham and radiation exposed animals treated with captopril or vehicle. There was no statistical difference between captopril or vehicle treated groups, so data was pooled for each group represented in histograms above. * indicates p values <0.05 as determined by 2-tailed, unpaired, t-tests. Error bars represent standard error of the mean (SEM). #for full length blots for each antibody, see Supplementary Fig. S11-S30

Supplementary Figure 9

GSK3β and pGSK3β expression in brain following total body radiation.

Histograms representing the densitometric ratio of levels of GSK3*β* (**A**.) and pGSK3*β* (**B**.) with respect to GAPDH as measured in the frontal cortex, hippocampus and cerebellum in the brains of Gottingen mini-pigs 30-days after total body radiation (1.79 Gy of Cobalt [^{60}Co]) with representative western blots# for sham and radiation exposed animals treated with captopril or vehicle. There was no statistical difference between captopril or vehicle treated groups, so data was pooled for each group represented in histograms above. *indicates p values <0.05 as

determined by 2-tailed, unpaired, *t*-tests. Error bars represent standard error of the mean (SEM). #for full length blots for each antibody, see Supplementary Fig. S11-S30

Supplementary Figure 10

PP2A-βα expression in brain following total body radiation.

Histograms representing the densitometric ratio of levels of PP2A-*βα* (**A**.) with respect to GAPDH as measured in the frontal cortex, hippocampus and cerebellum in the brains of Gottingen mini-pigs 30-days after total body radiation (1.79 Gy of Cobalt [^{60}Co]) with representative western blots# for sham and radiation exposed animals treated with captopril or vehicle. There was no statistical difference between captopril or vehicle treated groups, so data was pooled for each group represented in histograms above. *indicates *p* values <0.05 as determined by 2-tailed, unpaired, t-tests. Error bars represent standard error of the mean (SEM). #for full length blots for each antibody, see Supplementary Fig. S11-S30

Supplementary Figure 11

Images of the full-length blots for CP13, HT7 and βTubulin in Captopril and Vehicle Treated groups for Frontal Cortex and Hippocampus.

Supplementary Figure 12

Images of the full-length blots for CP13, HT7 and βTubulin in Captopril and Vehicle Treated groups for Cerebellum.

Supplementary Figure 13

Images of the full-length blots for APP, GAP43 and GFAP in Captopril and Vehicle Treated groups for Frontal Cortex and Hippocampus.

Supplementary Figure 14

Images of the full-length blots for APP, GAP43, and GFAP in Captopril and Vehicle Treated groups for Cerebellum.

Supplementary Figure 15

Images of the full-length blots for DNA Polymeraseβ, MBP and IBA1 in Captopril and Vehicle Treated groups for Frontal Cortex and Hippocampus.

Supplementary Figure 16

Images of the full-length blots for DNA Polymeraseβ, MBP and IBA1 in Captopril and Vehicle Treated groups for Cerebellum.

Supplementary Figure 17

Images of the full-length blots for GSK3β, pGSK3β, and PP2A-βα in Captopril and Vehicle Treated groups for Frontal Cortex and Hippocampus.

Supplementary Figure 18

Images of the full-length blots for GSK3β, pGSK3β, and PP2A-βα in Captopril and Vehicle Treated groups for Cerebellum.

Supplementary Figure 19

Images of full length GAPDH blot for CP13 primary antibody in Captopril and Vehicle treated groups for all 3 brain regions of interest. All blots were stripped one time and reprobed for GAPDH for normalization in densitometric analyses.

Supplementary Figure 20

Images of full length GAPDH blot for HT7 primary antibody in Captopril and Vehicle treated groups for all 3 brain regions of interest. All blots were stripped one time and reprobed for GAPDH for normalization in densitometric analyses.

Supplementary Figure 21

Images of full length GAPDH blot for βTubulin primary antibody in Captopril and Vehicle treated groups for all 3 brain regions of interest. All blots were stripped one time and reprobed for GAPDH for normalization in densitometric analyses.

Supplementary Figure 22

Images of full length GAPDH blot for APP primary antibody in Captopril and Vehicle treated groups for all 3 brain regions of interest. All blots were stripped one time and reprobed for GAPDH for normalization in densitometric analyses.

Supplementary Figure 23

Images of full length GAPDH blot for GAP43 primary antibody in Captopril and Vehicle treated groups for all 3 brain regions of interest. All blots were stripped one time and reprobed for GAPDH for normalization in densitometric analyses.

Supplementary Figure 24

Images of full length GAPDH blot for GFAP primary antibody in Captopril and Vehicle treated groups for all 3 brain regions of interest. All blots were stripped one time and reprobed for GAPDH for normalization in densitometric analyses.

Supplementary Figure 25

Images of full length GAPDH blot for DNA Polymeraseβ primary antibody in Captopril and Vehicle treated groups for all 3 brain regions of interest. All blots were stripped one time and reprobed for GAPDH for normalization in densitometric analyses.

Supplementary Figure 26

Images of full length GAPDH blot for MBP primary antibody in Captopril and Vehicle treated groups for all 3 brain regions of interest. All blots were stripped one time and reprobed for GAPDH for normalization in densitometric analyses.

Supplementary Figure 27

Images of full length GAPDH blot for IBA-1 primary antibody in Captopril and Vehicle treated groups for all 3 brain regions of interest. All blots were stripped one time and reprobed for GAPDH for normalization in densitometric analyses.

Supplementary Figure 28

Images of full length GAPDH blot for GSK3β primary antibody in Captopril and Vehicle treated groups for all 3 brain regions of interest. All blots were stripped one time and reprobed for GAPDH for normalization in densitometric analyses.

Supplementary Figure 29

Images of full length GAPDH blot for pGSK3β primary antibody in Captopril and Vehicle treated groups for all 3 brain regions of interest. All blots were stripped one time and reprobed for GAPDH for normalization in densitometric analyses.

Supplementary Figure 30

Images of full length GAPDH blot for PP2A-βα primary antibody in Captopril and Vehicle treated groups for all 3 brain regions of interest. All blots were stripped one time and reprobed for GAPDH for normalization in densitometric analyses.

Supplementary Figure 31

List of currently active or completed clinical trials using low-dose radiation therapies for dementia or Alzheimer's disease.
